# Exact soliton solutions and the significance of time-dependent coefficients in the Boussinesq equation: theory and application in mathematical physics

**DOI:** 10.1038/s41598-023-50782-1

**Published:** 2024-01-08

**Authors:** M. Abul Kawser, M. Ali Akbar, M. Ashrafuzzaman Khan, Hassan Ali Ghazwani

**Affiliations:** 1 Department of Mathematics, Islamic University, Kushtia, Bangladesh; 2 Department of Applied Mathematics, University of Rajshahi, Rajshahi, Bangladesh; 3 Department of Mechanical Engineering, College of Engineering, Jazan University, Jazan, Saudi Arabia

**Keywords:** Mathematics and computing, Physics

## Abstract

This article effectively establishes the exact soliton solutions for the Boussinesq model, characterized by time-dependent coefficients, employing the advanced modified simple equation, generalized Kudryashov and modified sine–Gordon expansion methods. The adaptive applicability of the Boussinesq system  to coastal dynamics, fluid behavior, and wave propagation enriches interdisciplinary research across hydrodynamics and oceanography. The solutions of the system obtained through these significant techniques make a path to understanding nonlinear phenomena in various fields, surpassing traditional barriers and further motivating research and application. Significant impacts of the coefficients of the equation, wave velocity, and related parameters are evident in the profiles of soliton-shaped waves in both 3D and 2D configurations when all these factors are treated as variables, which are not seen in the case for constant coefficients. This study enhances the understanding of the significant role played by nonlinear evolution equations with time-dependent coefficients through careful dynamic explanations and detailed analyses. This revelation opens up an interesting and challenging field of study, with promising insights that resonate across diverse scientific disciplines.

## Introduction

The variable coefficient nonlinear evolution equations (NLEEs) have become a pivotal and intricate realm of mathematical investigation, which forms a dynamic bridge between theoretical exploration and the intricacies of real-world phenomena^1,2^. These equations with variable coefficients play a vital role in modeling a wide array of natural systems, as they encapsulate the nuances that constant coefficients fail to capture^3,4^. By embodying spatial variability, NLEEs provide a richer understanding of complicated phenomena such as heat conduction, quantum mechanical interactions and fluid flow^5–7^. Also the equations underpin advancements in climate modeling, material science and medical imaging, guiding technological innovations by enhancing predictive capabilities and system understanding^8–10^. The implications of variable coefficient NLEEs extend beyond theoretical curiosity, permeating numerous scientific and engineering domains. In essence, the study of variable coefficient NLEEs reflects a harmonious combination between mathematical ingenuity and real-world complexity, accelerating our quest to decipher the fabric of the universe. In recent years, the search of numerical and exact solutions for nonlinear equations has gained particular prominence as a popular and captivating domain both in mathematics and physics. Various numerical methods contribute to the solution, such as Runge–Kutta method^11–13^, the Bayesian regularization technique (BRT)^14–17^, Levenberg–Marquardt approach (LMA)^18,19^, the shooting method^20–22^, the bvp4c technique^23–25^, the Keller box method^26–28^, the Lobatto IIIA method^29^, etc. Also researchers have developed several effective, powerful and efficient exact methods for uncovering the solutions of these equations. Notable techniques encompass the Jacobi elliptic function expansion scheme^30^, the Hirota bilinear method^31^, the exp-function method^32^, the new extended algebraic method^33^, the unified method^34^, the $F$-expansion technique^35^, the auxiliary equation outline^36^, the Darboux transformation technique^37^, the Bäcklund transformation^38^, the modified extended tanh technique with Riccati equation^39^, the generalized $(G^{\prime}/G)$-expansion approach^40^, the modified Kudryashov method^41–43^, the sine–Gordon expansion method^44^, the modified sine–cosine method^45^, the consistent Riccati expansion solvability technique^46^, the modified sine–Gordon expansion approach^47^, the modified simple equation method^48–50^, the generalized Kudryashov method^51–53^, among others.

The Boussinesq system is named after the French physicist Joseph Valentin Boussinesq involves a set of simplified equations derived from the Navier–Stokes equations in the nineteenth century, focusing on shallow water conditions and ignoring specific terms of less significance where the wavelength of waves is significantly larger than the water depth. This system finds applications in various fields such as tsunami modeling^54^, coastal engineering^55^, river/flood forecasting^56^, oceanography^57^, wave energy technology design^58^ and geophysical fluid dynamics^59^. It is utilized to predict wave effects, optimize energy devices, interpret internal wave behavior, forecast flows, simulate wave propagation and conduct studies in related areas^60–62^.

In this article, we deal with the Boussinesq equation, with a specific emphasis on its variable coefficients. A discussion of previous studies is necessary to gain a solid understanding of the nuances of this system. Wazwaz^63^ artfully applied the tanh method and the sine–cosine method, and Shakeel^64^ skillfully harnessed the novel $(G^{\prime}/G)$-expansion method to tackle the Boussinesq model with constant coefficients. Recently Chu et al.^65^ applied the Adomian decomposition method to handle this equation characterized by constant coefficients for fractional order. Notably, the present study endeavors to extend this understanding to the realm of variable coefficients, unveiling exact solutions through the adept employment of three specific techniques: the modified simple equation method, the generalized Kudryashov method and the modified sine–Gordon expansion method. The objective of the article is highlighted: to extend the understanding of the Boussinesq equations in the case of variable coefficients. The study uncovers the exact solutions using three specific techniques: the modified simple equation method, the generalized Kudryashov method, and the modified sine–Gordon expansion method. This extension builds upon previous works that primarily addressed the Boussinesq equation with constant coefficients, demonstrating the innovative approach of the present study in tackling the complexities of variable coefficients. The explanations of the obtained solutions show how the variable coefficients influence the system behavior and offering a detailed direction for comprehending the intricate interplay of nonlinear effects in a controlled manner. In this case, the behavior of wave shapes, amplitudes, wavelengths and propagation directions emerge as a dynamic interplay that continuously evolves across both space and time.

The rest of the article is organized in the following way: section "Analysis of the methods" provides a detailed description of the selected methods of research. In section "Extraction of solutions", solutions of the selected model are presented. section "Results and discussion" presents a graphical analysis of the obtained solutions. Finally, section “Conclusion” concludes the article, summarizing key insights and suggesting for future directions.

## Analysis of the methods

This section provides a broad overview of the employed methods to tackle the problem of variable coefficient Boussinesq system. In the investigation of wave solutions for the system, we used the power of three distinct yet complementary methods. Generally these techniques provide closed-form wave solutions with further free parameters than the other methods. Therefore, the obtained solutions can accurately explain the phenomenon of the system. Moreover, the chosen methods are able to provide all types of soliton solutions such as kink, periodic, bell-shape, spike type, and other solitons; which are not feasible in many techniques. So each of these approaches has been strategically chosen and meticulously executed to offer unique insights and solutions. Elaborations on the distinctive approaches of the three techniques in finding exact solutions are presented below.

### Common starting for the first two methods

Let us consider the evolution equation in the following nonlinear form:

1 $$F\left(u, {u}_{t}, {u}_{x}, {u}_{tt}, {u}_{xt}, {u}_{xx}, \dots \right)=0,$$

wherein $F$ is a polynomial function of $u(x, t)$ and its partial derivatives, incorporating both the highest order derivatives and nonlinear terms.

Consider the form below for the traveling wave transformation.

2 $$u\left(x, t\right)=U\left(\xi \right),\xi =p\left(t\right)x+q\left(t\right),$$

where both $p\left(t\right)$ and $q\left(t\right)$ are functions that possess differentiability with respect to $t$.

Through the application of this wave transformation, Eq. (1) can be transformed into the following nonlinear ordinary differential equation:

3 $$F\left(U, \left(\dot{p}x, \dot{q}\right){U}^{\prime}, p{U}^{\prime},{p}^{2} {U}^{{\prime}{\prime}}, p\left(\dot{p}x, \dot{q}\right){U}^{{\prime}{\prime}}, \dot{p}{U}^{\prime}, \dots \right)=0,$$

In here, the prime and dot marks signify the derivatives with respect to $\xi$ and $t$, respectively, $i.e. {U}^{\prime}=\frac{dU}{d\xi }, \dot{p}=\frac{dp}{dt}\mathrm{ and }\dot{q}=\frac{dq}{dt}$.

#### The modified simple equation method

Presented below are the major steps of the MSE method^48–50^:

Step 1: In accordance with the method, the solution $U(\xi )$ of Eq. (3) is regarded as:

4 $$U\left(\xi \right)={\sum }_{i=0}^{n}{a}_{i}\left(t\right){\left(\frac{{\varphi }^{\prime}\left(\xi \right)}{\varphi \left(\xi \right)}\right)}^{i}.$$

Each of the time-varying functions ${a}_{i}\left(t\right)$, $i=0, 1, 2, \dots , n$, where ${a}_{n}\left(t\right)\ne 0$, and the unidentified function $\varphi \left(\xi \right)$ must be determined, while $\varphi \left(\xi \right)$ satisfies both the conditions $\varphi \left(\xi \right)\ne 0$ and ${\varphi }^{\prime}\left(\xi \right)\ne 0$.

Step 2: By utilizing the balance homogeneity principle between the leading nonlinear terms and the maximum order derivatives present in Eq. (3), we determine the value of the positive integer $n$ appearing in Eq. (4).

Step 3: Inserting the sequential derivatives of $U\left(\xi \right)$ into Eq. (3) leads to the creation of a polynomial equation representing ${\varphi }^{-1}$. In the ensuing stages, a system of equations arises through the process of setting the coefficients of ${{x}^{k}\varphi }^{-i}$ to zero for $k=0, 1, 2, \dots$ and $i=0, 1, 2, \dots , n$ in the polynomial. This system of equations yields a collection of algebraic equations for the parameters ${a}_{i}\left(t\right), p\left(t\right), q\left(t\right)$ and differential equations involving the derivatives of $p\left(t\right), q\left(t\right)$ with regard to $t$.

Step 4: Subsequently, the solution of Eq. (3) can be found by first solving the system derived in step 3 to determine $\varphi \left(\xi \right), {a}_{i}\left(t\right), p\left(t\right)$ and $q\left(t\right)$, and then substituting these values into Eq. (4), ultimately leading to the solutions of Eq. (1).

#### The Generalized Kudryashov method

Following are the key steps that describe the methodology of the generalized Kudryashov^51–53^:

Step 1: The approach proposes that the precise solution for Eq. (1) be represented in the subsequent rational structure:

5 $$U\left(\xi \right)=\frac{\sum_{i=0}^{n}{a}_{i}(t){\left(Q\left(\xi \right)\right)}^{i}}{\sum_{j=0}^{m}{b}_{j}(t){\left(Q\left(\xi \right)\right)}^{j}},$$

where ${a}_{i}\left(t\right) (i=0, 1, 2, \dots , n)$ and ${b}_{j}\left(t\right) (j=0, 1, 2, \dots , m)$ are functions dependent on t, to be solved at a later juncture, under the conditions ${a}_{n}\left(t\right)\ne 0$ and ${b}_{m}\left(t\right)\ne 0$. Furthermore, the following ODE is satisfied by $Q(\xi )$:

6 $${Q}^{\prime}\left(\xi \right)=Q\left(\xi \right)\left(Q\left(\xi \right)-1\right).$$

It is clear that Eq. (6) possesses a solution in the following form:

7 $$Q\left(\xi \right)=\frac{1}{1+A{e}^{\xi }}.$$

Here, the symbol $A$ stands as an integrating constant.

Step 2: The principle of homogeneous balance will guide us to identify the choices of positive integer for $n$ and $m$ within Eq. (5). To illustrate, we achieve balance by equating the highest order derivative with the corresponding highest order nonlinear term presented in Eq. (3).

Step 3: Substituting Eq. (5) into Eq. (3) along with Eq. (6), we acquire a polynomial of $Q(\xi )$. By setting all of the coefficients of the like powers of $Q(\xi )$, which are multiplied by ${x}^{k}$ for $k=0, 1, 2, \dots$ to zero, we attain both algebraic and differential systems of equations of parameters ${a}_{i}$, ${b}_{j}$, $p$, $q$ and $p$, $q$ respectively. Through the utilization of the Mathematica or Maple software package program, these systems of equations can be solved. This process enables us to compute the values of the unknown parameters ${a}_{i}$, ${b}_{j}$, $p$ and $q$. Subsequently, we find the exact solutions for the reduced Eq. (3).

### The modified sine–Gordon expansion method

The sine–Gordon equation is given by

8 $${u}_{xx}-{u}_{tt}={\text{sin}}\left(u\right).$$

Herein, $u$ denotes a function of both $x$ and $t$. By introducing the wave variable $\xi =px+qt$, we can rewrite the preceding equation as the subsequent nonlinear expression.

9 $$U^{\prime}{\prime}=\left\{1/({p}^{2}+{q}^{2})\right\}{\text{sin}}\left(U\right).$$

Here, the variable $U\left(\xi \right)=u(x, t)$, where $\xi$ and $q/p$ stand for the amplitude and velocity of the traveling wave, respectively. Now Eq. (9) can be expressed as a first order differential equation as follows:

10 $${(U^{\prime}/2)}^{2}=\left\{1/({p}^{2}+{q}^{2})\right\}{{\text{sin}}}^{2}(U/2)+K.$$

The $K$ appears in the above equation as an integrating constant. Substituting $U(\xi )/2=w(\xi )$, ${a}^{2}=1/({p}^{2}+{q}^{2})$ and $K=0$ into Eq. (10) yields:

11 $${w}^{\prime}=\frac{dw}{d\xi }={\text{sin}}\left(w\right),$$

taking $$w=w(\xi )$$ and $$a=1$$.

By solving Eq. (11) through separation of variables and simplification, we can confirm the validity of the following two interesting relations:

12 $${\text{sin}}\left(w\right)={\text{sin}}\left(w\left(\xi \right)\right)={\left.\frac{2r{e}^{\xi }}{{r}^{2}{e}^{2\xi }+1}\right]}_{r=1}={\text{sech}}\left(\xi \right),$$

13 $$\mathrm{and}\; \mathrm{cos}\left(w\right)={\text{cos}}\left(w\left(\xi \right)\right)={\left.\frac{{r}^{2}{e}^{2\xi }-1}{{r}^{2}{e}^{2\xi }+1}\right]}_{r=1}={\text{tanh}}\left(\xi \right).$$

The value of the integrating constant, $r\ne 0$.

In light of the assumption, the solution for the polynomial nonlinear wave equation having variable coefficients of the form $F\left(u, {u}_{t}, {u}_{x}, {u}_{tt}, {u}_{xt}, {u}_{xx}, \dots \right)=0$ can be regarded as:

14 $$U\left(\xi \right)=\sum_{i=1}^{n}{{\text{tanh}}}^{i-1}(\xi )\left\{{b}_{i}\left(t\right){\text{sech}}\left(\xi \right)+{a}_{i}\left(t\right){\text{tanh}}(\xi )\right\}+{a}_{0}\left(t\right).$$

The transformation $U\left(\xi \right)=u(x, t)$, $\xi =p\left(t\right)x+q(t)$ is utilized to reformulate the nonlinear wave equation with time-dependent coefficients, leading to an equivalent nonlinear equation, with $p, q, {a}_{0}, {a}_{i}$ and ${b}_{i}$ $(i=1, 2, 3, \dots , n)$ as functions of time.

Through the application of the results indicated in Eqs. (12) and (13), we can represent Eq. (14) in an alternative form as follows:

15 $$U\left(w\right)=\sum_{i=1}^{n}{{\text{cos}}}^{i-1}(w)\left\{{b}_{i}\left(t\right){\text{sin}}(w)+{a}_{i}\left(t\right){\text{cos}}(w)\right\}+{a}_{0}\left(t\right),$$

which represents a polynomial of degree $n$.

The parameter $n$ can be determined using the balance procedure, which is the comparison of the highest order derivative with the highest nonlinear term. Subsequent to finding the parameter $n$, simplifying by inserting solution (15) into the transformed nonlinear equation yields a trigonometric series in the form of ${x}^{k}{\text{sin}}(jw)$ and ${x}^{k}{\text{cos}}(jw)$, where $j=0, 1, 2, \dots , n$ and $k=0, 1, 2, \dots$^47^. By equating to zero all the relevant harmonic terms ${\text{sin}}(jw)$ and ${\text{cos}}(jw)$ in multiplicative form with ${x}^{k}$, we establish a set of equations that relate to the unknowns $p, q, {a}_{0}, {a}_{i}$ and ${b}_{i}$. As a result, the problem converts into algebraic equations for $p, q, {a}_{0}, {a}_{i}, {b}_{i}$ and differential equations of $p$ and $q$ in relation to time. Computerized calculations are used to ascertain the values of $p, q, {a}_{0}, {a}_{i}, {b}_{i}$ and subsequently, the solutions proposed in Eqs. (12), (13) and (15) are applied.

## Extraction of solutions

Consider the Boussinesq model subject to the variable coefficients of the following form:

16 $${u}_{t}+{v}_{x}=0,$$

17 $${v}_{t}+\alpha (t){\left({u}^{2}\right)}_{x}-\beta (t){u}_{xxx}=0,$$

with $\alpha (t)$ and $\beta (t)$ being differentiable functions throughout all $t$.

The system of equations stated above is utilized to model the bidirectional movement of particular water waves in a smooth, horizontal channel saturated with a liquid that is both inviscid and irrotational^66^. The time-dependent coefficients in the model indicate that the properties of the medium or fluid are changing with time. These coefficients can significantly influence the transmission of waves through the medium, leading to multifaceted wave interactions and phenomena.

By implementing the transformations $U(\xi )=u(x, t)$ and $V(\xi )=v(x, t)$, with $\xi$ representing the wave variable defined as $\xi =p(t)x+q(t)$, and substituting them into Eqs. (16) and (17), the equations transform as follows:

18 $$\left(x\dot{p}+\dot{q}\right)U^{\prime}+pV^{\prime}=0,$$

19 $$\left(x\dot{p}+\dot{q}\right)V^{\prime}+2\alpha pUU^{\prime}-\beta {p}^{3}U^{{\prime}{\prime}{\prime}}=0,$$

Integrating once Eqs. (18) and (19) with regard to $\xi$, we obtain

20 $$V = \frac{{\left( {x\dot{p} + \dot{q}} \right)U}}{p}.$$

21 $$\left(x\dot{p}+\dot{q}\right)V+p{U}^{2}\alpha -{p}^{3}\beta U^{{\prime}{\prime}}=0,$$

By replacing $V$ from Eq. (20) into Eq. (21) and simplifying thereafter, we derive

22 $${p}^{4}\beta {U}^{{\prime}{\prime}}+{\left(x\dot{p}+\dot{q}\right)}^{2}U-{p}^{2}\alpha {U}^{2}=0,$$

The utilization of the homogeneous balance principle for the highest order linear term $U^{{\prime}{\prime}}$ and the nonlinear term ${U}^{2}$ in (22) results in

$$n+2=2n,\mathrm{ implies }\quad n=2.$$

### Solutions through the modified simple equation method

Since the balance number is $n=2$, so we will explore the solution for Eq. (22) in accordance with the following prescribed structure of the method:

23 $$U\left(\xi \right)={a}_{0}\left(t\right)+{a}_{1}\left(t\right)\left(\frac{{\varphi }^{{\prime}}\left(\xi \right)}{\varphi \left(\xi \right)}\right)+{a}_{2}\left(t\right){\left(\frac{{\varphi }^{{\prime}}\left(\xi \right)}{\varphi \left(\xi \right)}\right)}^{2}.$$

As the method dictates, substituting (23) into (22) yields the following nonlinear algebraic and differential equations:

24 $$\begin{gathered} p^{2} \alpha a_{0}^{2} - a_{0} \dot{q}^{2} = 0,a_{0} \dot{p}\dot{q} = 0,a_{0} \dot{p}^{2} = 0,\left( {2p^{2} \alpha a_{0} a_{1} - a_{1} \dot{q}^{2} } \right)\varphi^{\prime } - p^{4} \beta a_{1} \varphi^{\prime \prime \prime } = 0, \hfill \\ a_{1} \dot{p}\dot{q}\varphi^{\prime } = 0,a_{1} \dot{p}^{2} \varphi^{\prime } = 0,a_{2} \dot{p}\dot{q}\varphi^{\prime 2} = 0a_{2} \dot{p}^{2} \varphi^{\prime 2} = 0, \hfill \\ \left( {p^{2} \alpha \left( {a_{1}^{2} + 2a_{0} a_{2} } \right) - a_{2} \dot{q}^{2} } \right)\varphi^{\prime 2} + p^{4} \beta \left( {3a_{1} \varphi^{\prime } \varphi^{\prime \prime } - 2a_{2} \left( {\varphi^{^{\prime\prime}2} + \varphi^{\prime } \varphi^{\prime \prime \prime } } \right)} \right) = 0, \hfill \\ \left( {p^{4} \beta a_{1} - p^{2} \alpha a_{1} a_{2} } \right)\varphi^{\prime 3} - 5p^{4} \beta a_{2} \varphi^{\prime 2} \varphi^{\prime \prime } = 0, \hfill \\ \left( {6p^{4} \beta a_{2} - p^{2} \alpha a_{2}^{2} } \right)\varphi^{\prime 4} = 0, \hfill \\ \end{gathered}$$

Solving the above set of algebraic and differential equations by using Mathematica software package program, we acquire solutions which are representing below:

$${a}_{0}=\frac{\alpha {a}_{1}^{2}}{36{\lambda }^{2}\beta },{a}_{1}={a}_{1},{a}_{2}=\frac{6{\lambda }^{2}\beta }{\alpha },p=\lambda ,q=\mu \pm \frac{1}{6}\int \frac{\alpha {a}_{1}}{\sqrt{\beta }}dt\; \mathrm{ and }\; \; \varphi =B-\left(\frac{6{\lambda }^{2}A\beta }{\alpha {a}_{1}}\right){e}^{-\left(\frac{\alpha {a}_{1}}{6{\lambda }^{2}\beta }\right)\xi }.$$

Thus, the variable coefficient Boussinesq system yields the following exact traveling wave solutions:

25 $$U\left(\xi \right)=\frac{\alpha {a}_{1}^{2}}{36{\lambda }^{2}\beta }+\frac{AB{\alpha }^{2}{a}_{1}^{3}{e}^{\left(\frac{\alpha {a}_{1}}{6{\lambda }^{2}\beta }\right)\xi }}{{\left(-6A{\lambda }^{2}\beta +B\alpha {a}_{1}{e}^{\left(\frac{\alpha {a}_{1}}{6{\lambda }^{2}\beta }\right)\xi }\right)}^{2}},$$

26 $$V\left(\xi \right)= -\frac{\alpha {a}_{1}^{2}\dot{q}}{36{\lambda }^{3}\beta }-\frac{AB{\alpha }^{2}{a}_{1}^{3}\dot{q}{e}^{\left(\frac{\alpha {a}_{1}}{6{\lambda }^{2}\beta }\right)\xi }}{{\lambda \left(-6A{\lambda }^{2}\beta +B\alpha {a}_{1}{e}^{\left(\frac{\alpha {a}_{1}}{6{\lambda }^{2}\beta }\right)\xi }\right)}^{2}}.$$

The above mentioned solutions can be transformed into hyperbolic functions, represented as:

27 $$U\left(\xi \right)=\frac{\alpha {a}_{1}^{2}}{36{\lambda }^{2}\beta }+\frac{AB{\alpha }^{2}{a}_{1}^{3}}{{\left(\left(B\alpha {a}_{1}-6A{\lambda }^{2}\beta \right){\text{cosh}}\left(\frac{\alpha {a}_{1}\xi }{12{\lambda }^{2}\beta }\right)+\left(B\alpha {a}_{1}+6A{\lambda }^{2}\beta \right){\text{sinh}}\left(\frac{\alpha {a}_{1}\xi }{12{\lambda }^{2}\beta }\right)\right)}^{2}},$$

28 $$V\left(\xi \right)=-\frac{\alpha {a}_{1}^{2}\dot{q}}{36{\lambda }^{3}\beta }-\frac{AB{\alpha }^{2}{a}_{1}^{3}\dot{q}}{\lambda {\left(\left(B\alpha {a}_{1}-6A{\lambda }^{2}\beta \right){\text{cosh}}\left(\frac{\alpha {a}_{1}\xi }{12{\lambda }^{2}\beta }\right)+\left(B\alpha {a}_{1}+6A{\lambda }^{2}\beta \right){\text{sinh}}\left(\frac{\alpha {a}_{1}\xi }{12{\lambda }^{2}\beta }\right)\right)}^{2}}.$$

As $A$ and $B$ are arbitrary functions of time. So it is possible to select their values arbitrarily, which are producing a variety of potential outcomes:

**Case I:** Assuming $A=-\frac{\alpha {a}_{1}}{{\lambda }^{2}\beta }$ and $B=6$, then solution (27) and (28) takes the form:

29 $$U\left(\xi \right)=\frac{\alpha {a}_{1}^{2}}{12{\lambda }^{2}\beta }\left(\frac{1}{3}-\frac{1}{2}{{\text{sech}}}^{2}\left(\frac{\alpha {a}_{1}\xi }{12{\lambda }^{2}\beta }\right)\right),$$

30 $$V\left(\xi \right)=-\frac{\alpha {a}_{1}^{2}\dot{q}}{12{\lambda }^{3}\beta }\left(\frac{1}{3}-\frac{1}{2}{{\text{sech}}}^{2}\left(\frac{\alpha {a}_{1}\xi }{12{\lambda }^{2}\beta }\right)\right).$$

**Case II:** The choice $A=\frac{1}{{\lambda }^{2}\beta }$ and $B=\frac{1}{\alpha {a}_{1}}$ convert the solutions (27) and (28) into the form:

31 $$U\left(\xi \right)=\frac{\alpha {a}_{1}^{2}}{36{\lambda }^{2}\beta }+\frac{\alpha {a}_{1}^{2}}{{\lambda }^{2}\beta {\left(5{\text{cosh}}\left(\frac{\alpha {a}_{1}\xi }{12{\lambda }^{2}\beta }\right)-7{\text{sinh}}\left(\frac{\alpha {a}_{1}\xi }{12{\lambda }^{2}\beta }\right)\right)}^{2}},$$

32 $$V\left(\xi \right)=-\frac{\alpha {a}_{1}^{2}\dot{q}}{36{\lambda }^{3}\beta }-\frac{\alpha {a}_{1}^{2}\dot{q}}{{\lambda }^{3}\beta {\left(5{\text{cosh}}\left(\frac{\alpha {a}_{1}\xi }{12{\lambda }^{2}\beta }\right)-7{\text{sinh}}\left(\frac{\alpha {a}_{1}\xi }{12{\lambda }^{2}\beta }\right)\right)}^{2}}.$$

**Case III:** If we consider $A=\frac{1}{{6\lambda }^{2}}$ and $B=\frac{1}{{a}_{1}}$ then solution (28) and (28) becomes

33 $$U\left(\xi \right)=\frac{\alpha {a}_{1}^{2}}{36{\lambda }^{2}\beta }+\frac{{\alpha }^{2}{a}_{1}^{2}}{6{\lambda }^{2}{\left(\left(\alpha -\beta \right){\text{cosh}}\left(\frac{\alpha {a}_{1}\xi }{12{\lambda }^{2}\beta }\right)+\left(\alpha +\beta \right){\text{sinh}}\left(\frac{\alpha {a}_{1}\xi }{12{\lambda }^{2}\beta }\right)\right)}^{2}},$$

34 $$V\left(\xi \right)=-\frac{\alpha {a}_{1}^{2}\dot{q}}{36{\lambda }^{3}\beta }-\frac{{\alpha }^{2}{a}_{1}^{2}\dot{q}}{6{\lambda }^{3}{\left(\left(\alpha -\beta \right){\text{cosh}}\left(\frac{\alpha {a}_{1}\xi }{12{\lambda }^{2}\beta }\right)+\left(\alpha +\beta \right){\text{sinh}}\left(\frac{\alpha {a}_{1}\xi }{12{\lambda }^{2}\beta }\right)\right)}^{2}}.$$

**Case IV:** Setting $A=\frac{\alpha {a}_{1}}{{\lambda }^{2}\beta }$ and $B=6$ in solutions (27) and (28), then

35 $$U\left(\xi \right)=\frac{\alpha {a}_{1}^{2}}{12{\lambda }^{2}\beta }\left(\frac{1}{3}+\frac{1}{2}{{\text{csch}}}^{2}\left(\frac{\alpha {a}_{1}\xi }{12{\lambda }^{2}\beta }\right)\right),$$

36 $$V\left(\xi \right)=-\frac{\alpha {a}_{1}^{2}\dot{q}}{12{\lambda }^{3}\beta }\left(\frac{1}{3}+\frac{1}{2}{{\text{csch}}}^{2}\left(\frac{\alpha {a}_{1}\xi }{12{\lambda }^{2}\beta }\right)\right).$$

wherein $\xi =p\left(t\right)x+q(t)$ with $p\left(t\right)=\lambda$ and $q=\mu \pm \frac{1}{6}\int \frac{\alpha {a}_{1}}{\sqrt{\beta }}dt$.

In the same process, for other considerations of the integral constants $A$ and $B$, more important and logical wave solutions can be obtained but the solutions are not shown in order to keep the size of this article to an elegant volume.

### Solutions through the generalized Kudryashov method

Applying the homogeneous balance principle to the leading linear term $U^{{\prime}{\prime}}$ and the non-linear term ${U}^{2}$ in Eq. (22) yields:

$$n-m+2=2n-2m,\mathrm{ implies }n=m+2.$$

If we choose $m=1$, which gives $n=3$.

Thus according to the method, the exact solution of (22) takes the following form:

37 $$U\left(\xi \right)=\frac{{a}_{0}\left(t\right)+{a}_{1}\left(t\right)Q\left(\xi \right)+{a}_{2}\left(t\right){\left(Q\left(\xi \right)\right)}^{2}+{a}_{3}\left(t\right){\left(Q\left(\xi \right)\right)}^{3}}{{b}_{0}\left(t\right)+{b}_{1}\left(t\right)Q\left(\xi \right)}.$$

Substituting (37) into (22) and using relation ${Q}^{{\prime}}\left(\xi \right)=Q(\xi )(Q\left(\xi \right)-1)$, we derive the subsequent nonlinear algebraic and differential equations following the method

38 $$\begin{aligned} & p^{2} \alpha a_{0}^{2} b_{0} - a_{0} b_{0}^{2} \dot{q}^{2} = 0, a_{0} b_{0}^{2} \dot{p}\dot{q} = 0, a_{0} b_{0}^{2} \dot{p}^{2} = 0, \left( {a_{1} b_{0}^{2} + 2a_{0} b_{0} b_{1} } \right)\dot{p}\dot{q} = 0, \\ & \quad p^{2} \alpha \left( {2a_{0} a_{1} b_{0} + a_{0}^{2} b_{1} } \right) - p^{4} \beta \left( {a_{1} b_{0}^{2} - a_{0} b_{0} b_{1} } \right) - \left( {a_{1} b_{0}^{2} + 2a_{0} b_{0} b_{1} } \right)\dot{q}^{2} = 0, \\ & \quad \left( {a_{1} b_{0}^{2} + 2a_{0} b_{0} b_{1} } \right)\dot{p}^{2} = 0,\left( {a_{2} b_{0}^{2} + 2a_{1} b_{0} b_{1} + a_{0} b_{1}^{2} } \right)\dot{p}\dot{q} = 0, \\ & \quad p^{2} \alpha (a_{1}^{2} b_{0} + 2a_{0} a_{2} b_{0} + 2a_{0} a_{1} b_{1} ) + p^{4} \beta (3a_{1} b_{0}^{2} - 4a_{2} b_{0}^{2} \\ & \quad - 3a_{0} b_{0} b_{1} + a_{1} b_{0} b_{1} - a_{0} b_{1}^{2} ) - \left( {a_{2} b_{0}^{2} + 2a_{1} b_{0} b_{1} + a_{0} b_{1}^{2} } \right)\dot{q}^{2} = 0, \\ & \quad \left( {a_{2} b_{0}^{2} + 2a_{1} b_{0} b_{1} + a_{0} b_{1}^{2} } \right)\dot{p}^{2} = 0, \\ & \quad p^{2} \alpha \left( {2b_{0} \left( {a_{1} a_{2} + a_{0} a_{3} } \right) + b_{1} (a_{1}^{2} + 2a_{0} a_{2} } \right)) - p^{4} \beta (b_{0}^{2} \left( {2a_{1} - 10a_{2} + 9a_{3} } \right) \\ & \quad - b_{1} \left( {2a_{0} b_{0} - a_{1} b_{0} - 3a_{2} b_{0} + a_{0} b_{1} } \right)) - \left( {a_{3} b_{0}^{2} + 2a_{2} b_{0} b_{1} + a_{1} b_{1}^{2} } \right)\dot{q}^{2} = 0, \\ & \quad \left( {a_{3} b_{0}^{2} + 2a_{2} b_{0} b_{1} + a_{1} b_{1}^{2} } \right)\dot{p}\dot{q} = 0,\left( {a_{3} b_{0}^{2} + 2a_{2} b_{0} b_{1} + a_{1} b_{1}^{2} } \right)\dot{p}^{2} = 0, \\ & \quad p^{2} \alpha \left( {a_{2}^{2} b_{0} + 2a_{1} a_{3} b_{0} + 2a_{1} a_{2} b_{1} + 2a_{0} a_{3} b_{1} } \right) - p^{4} \beta (6a_{2} b_{0}^{2} \\ & \quad - 21a_{3} b_{0}^{2} - 9a_{2} b_{0} b_{1} + 11a_{3} b_{0} b_{1} + a_{2} b_{1}^{2} ) - \left( {2a_{3} b_{0} b_{1} + a_{2} b_{1}^{2} } \right)\dot{q}^{2} = 0, \\ & \quad \left( {2a_{3} b_{0} b_{1} + a_{2} b_{1}^{2} } \right)\dot{p}\dot{q} = 0,\left( {2a_{3} b_{0} b_{1} + a_{2} b_{1}^{2} } \right)\dot{p}^{2} = 0, \\ & \quad p^{2} \alpha (2a_{2} a_{3} b_{0} + a_{2}^{2} b_{1} + 2a_{1} a_{3} b_{1} ) - p^{4} \beta (12a_{3} b_{0}^{2} + 6a_{2} b_{0} b_{1} \\ & \quad - 27a_{3} b_{0} b_{1} - 3a_{2} b_{1}^{2} + 4a_{3} b_{1}^{2} ) - a_{3} b_{1}^{2} \dot{q}^{2} = 0,a_{3} b_{1}^{2} \dot{p}\dot{q} = 0,a_{3} b_{1}^{2} \dot{p}^{2} = 0, \\ & \quad p^{2} \alpha \left( {a_{3}^{2} b_{0} + 2a_{2} a_{3} b_{1} } \right) - 2p^{4} \beta \left( {8a_{3} b_{0} b_{1} + a_{2} b_{1}^{2} - 5a_{3} b_{1}^{2} } \right) = 0, \\ & \quad p^{2} \alpha a_{3}^{2} b_{1} - 6p^{4} \beta a_{3} b_{1}^{2} = 0, \\ \end{aligned}$$

Solving the above set of algebraic and differentials presented in Eqs. (38), using Mathematica software, we found solutions that are presented below:

$${a}_{0}=\frac{{\uplambda }^{2}\beta {b}_{0}}{\alpha },{a}_{1}=\frac{{\uplambda }^{2}\beta (-6{b}_{0}+{b}_{1})}{\alpha },{a}_{2}=\frac{6{\uplambda }^{2}\beta ({b}_{0}-{b}_{1})}{\alpha },{a}_{3}=\frac{6{\uplambda }^{2}\beta {b}_{1}}{\alpha },{b}_{0}={b}_{0},{b}_{1}={b}_{1},p=\lambda \mathrm{ and }q=\mu \pm {\uplambda }^{2}\int \sqrt{\beta }dt.$$

Hence by employing the generalized Kudryashov method, we obtain the solutions for the Boussinesq system with variable coefficients (14) as follows:

39 $$U\left(\xi \right)=\frac{(1-4A{e}^{\xi }+{A}^{2}{e}^{2\xi }){\lambda }^{2}\beta }{{(1+A{e}^{\xi })}^{2}\alpha },$$

40 $$V\left(\xi \right)=-\frac{\left(1-4A{e}^{\xi }+{A}^{2}{e}^{2\xi }\right)\lambda \beta \dot{q}}{{\left(1+A{e}^{\xi }\right)}^{2}\alpha },$$

where $A$ stands as an arbitrary constant.

The solutions described above can be expressed in terms of hyperbolic functions as follows:

41 $$U\left(\xi \right)=\frac{{\lambda }^{2}\beta \left(-4A+\left({A}^{2}+1\right){\text{cosh}}\xi +\left({A}^{2}-1\right){\text{sinh}}\xi \right)}{\alpha {\left(\left(A+1\right){\text{cosh}}\left(\frac{\xi }{2}\right)+\left(A-1\right){\text{sinh}}\left(\frac{\xi }{2}\right)\right)}^{2}},$$

42 $$V\left(\xi \right)=-\frac{\lambda \beta \dot{q}\left(-4A+\left({A}^{2}+1\right){\text{cosh}}\xi +\left({A}^{2}-1\right){\text{sinh}}\xi \right)}{\alpha {\left(\left(A+1\right){\text{cosh}}\left(\frac{\xi }{2}\right)+\left(A-1\right){\text{sinh}}\left(\frac{\xi }{2}\right)\right)}^{2}}.$$

**Case I**: If we choose $A=1$ in the solutions (41) and (42), then

43 $$U\left(\xi \right)=\frac{{\lambda }^{2}\beta ({\text{cosh}}\xi -2)}{\alpha ({\text{cosh}}\xi +1)},$$

44 $$V\left(\xi \right)=-\frac{\lambda \beta \dot{q}\left({\text{cosh}}\xi -2\right)}{\alpha \left({\text{cosh}}\xi +1\right)}.$$

**Case II**: The consideration $A=2$ converts the solution (41) and (42) into the form:

45 $$U\left(\xi \right)=\frac{{\lambda }^{2}\beta (-8+5{\text{cosh}}\xi +3{\text{sinh}}\xi )}{\alpha (4+5{\text{cosh}}\xi +3{\text{sinh}}\xi )},$$

46 $$V\left(\xi \right)=-\frac{\lambda \beta \dot{q}\left(-8+5{\text{cosh}}\xi +3{\text{sinh}}\xi \right)}{\alpha \left(4+5{\text{cosh}}\xi +3{\text{sinh}}\xi \right)}.$$

**Case III**: If we set $A=-2$, then the solution (41) and (42) becomes

47 $$U\left(\xi \right)=\frac{{\lambda }^{2}\beta (8+5{\text{cosh}}\xi +3{\text{sinh}}\xi )}{\alpha (-4+5{\text{cosh}}\xi +3{\text{sinh}}\xi )},$$

48 $$V\left(\xi \right)=-\frac{\lambda \beta \dot{q}\left(8+5{\text{cosh}}\xi +3{\text{sinh}}\xi \right)}{\alpha \left(-4+5{\text{cosh}}\xi +3{\text{sinh}}\xi \right)}.$$

**Case IV**: Suppose $A=-1$, then the solution (41) and (42) takes the form:

49 $$U\left(\xi \right)=\frac{{\lambda }^{2}\beta ({\text{cosh}}\xi +2)}{\alpha ({\text{cosh}}\xi -1)},$$

50 $$V\left(\xi \right)=-\frac{\lambda \beta \dot{q}\left({\text{cosh}}\xi +2\right)}{\alpha \left({\text{cosh}}\xi -1\right)}.$$

Here $\xi =p\left(t\right)x+q(t)$, where $p\left(t\right)=\lambda$ and $q=\mu \pm {\uplambda }^{2}\int \sqrt{\beta }dt$.

By similar process, it is possible to derive numerous other indispensable and consistent wave solutions through variations in the integral constants $A$. Nonetheless, in order to maintain elegant brevity of the paper we have chosen not to include these solutions.

### Solutions through the modified sine–Gordon expansion method

As the balance number, $n=2$. So based on the method, consider the solution for Eq. (22):

51 $$U\left(w\right)={b}_{2}{\text{sin}}(w){\text{cos}}(w)+{b}_{1}{\text{sin}}\left(w\right)+{a}_{2}{{\text{cos}}}^{2}\left(w\right)+{a}_{1}{\text{cos}}\left(w\right)+{a}_{0}.$$

By substituting (51) into (22) together with ${w}^{\prime}\left(\xi \right)={\text{sin}}(w)$, then as per the method we can derive the following nonlinear algebraic and differential equations:

52 $$\begin{aligned} & p^{2} \alpha \left( {4a_{0} b_{1} + a_{2} b_{1} + a_{1} b_{2} } \right) + p^{4} \beta b_{1} - 2b_{1} \dot{q}^{2} = 0,b_{1} \dot{p}\dot{q} = 0,b_{1} \dot{p}^{2} = 0, \\ & \quad p^{2} \alpha \left( {4a_{0} a_{1} + 3a_{1} a_{2} + b_{1} b_{2} } \right) + p^{4} \beta a_{1} - 2a_{1} \dot{q}^{2} = 0, \\ & \quad a_{1} \dot{p}\dot{q} = 0,a_{1} \dot{p}^{2} = 0,p^{2} \alpha \left( {2a_{1} b_{1} + 2a_{0} b_{2} + a_{2} b_{2} } \right) + 2p^{4} \beta b_{2} - b_{2} \dot{q}^{2} = 0, \\ & \quad b_{2} \dot{p}\dot{q} = 0,b_{2} \dot{p}^{2} = 0,p^{2} \alpha \left( {a_{1}^{2} + 2a_{0} a_{2} + a_{2}^{2} - b_{1}^{2} } \right) + 2p^{4} \beta a_{2} - a_{2} \dot{q}^{2} = 0, \\ & \quad a_{2} \dot{p}\dot{q} = 0,a_{2} \dot{p}^{2} = 0,p^{2} \alpha \left( {a_{2} b_{1} + a_{1} b_{2} } \right) - p^{4} \beta b_{1} = 0, \\ & \quad p^{2} \alpha \left( {a_{1} a_{2} - b_{1} b_{2} } \right) - p^{4} \beta a_{1} = 0,3p^{4} \beta b_{2} - p^{2} \alpha a_{2} b_{2} = 0, \\ & \quad p^{2} \alpha \left( {a_{2}^{2} - b_{2}^{2} } \right) - 6p^{4} \beta a_{2} = 0, \\ & \quad p^{2} \alpha \left( {8a_{0}^{2} + 4a_{1}^{2} + 8a_{0} a_{2} + 3a_{2}^{2} + 4b_{1}^{2} + b_{2}^{2} } \right) - 2p^{4} \beta a_{2} - 4\left( {2a_{0} + a_{2} } \right)\dot{q}^{2} = 0, \\ & \quad \left( {2a_{0} + a_{2} } \right)\dot{p}\dot{q} = 0, \\ & \quad \left( {2a_{0} + a_{2} } \right)\dot{p}^{2} = 0, \\ \end{aligned}$$

By using the Mathematica software package program to solve the system of algebraic and differential Eqs. (52) presented above, we obtain different sets of solutions that are presented below:

Set 1: $${a}_{0}=-\frac{6{\lambda }^{2}\beta }{\alpha }$$, $${a}_{1}=0$$, $${a}_{2}=\frac{6{\lambda }^{2}\beta }{\alpha }$$, $${b}_{1}=0$$, $${b}_{2}=0$$, $$p=\lambda$$, $$q=\mu \pm 2i{\lambda }^{2}\int \sqrt{\beta }dt$$.

By using these results, the modified sine–Gordon expansion approach provides the solutions of the time-varying coefficient Boussinesq model as follows:

53 $$U\left(\xi \right)=-\frac{6{\lambda }^{2}\beta {{\text{sech}}}^{2}\xi }{\alpha },$$

54 $$V\left(\xi \right)=\frac{6\lambda \beta \dot{q}{{\text{sech}}}^{2}\xi }{\alpha }.$$

Set 2: $${a}_{0}=-\frac{2{\lambda }^{2}\beta }{\alpha }$$, $${a}_{1}=0$$, $${a}_{2}=\frac{6{\lambda }^{2}\beta }{\alpha }$$, $${b}_{1}=0$$, $${b}_{2}=0$$, $$p=\lambda$$, $$q=\mu \pm 2{\lambda }^{2}\int \sqrt{\beta }dt$$.

The technique, with the aid of these results, offers the solutions to the time-varying coefficient Boussinesq system as follows:

55 $$U\left(\xi \right)=\frac{2{\lambda }^{2}\beta (-1+3{{\text{tanh}}}^{2}\xi )}{\alpha },$$

56 $$V\left(\xi \right)=\frac{2\lambda \beta \dot{q}(1-3{{\text{tanh}}}^{2}\xi )}{\alpha }.$$

Set 3: $${a}_{0}=-\frac{3{\lambda }^{2}\beta }{\alpha }$$, $${a}_{1}=0$$, $${a}_{2}=\frac{3{\lambda }^{2}\beta }{\alpha }$$, $${b}_{1}=0$$, $${b}_{2}=\pm \frac{3i{\lambda }^{2}\beta }{\alpha }$$, $$p=\lambda$$, $$q=\mu \pm i{\lambda }^{2}\int \sqrt{\beta }dt$$.

These results enable the modified sine–Gordon expansion method to present the solutions of the time-varying coefficient Boussinesq equations as follows:

57 $$U\left(\xi \right)=\frac{3{\lambda }^{2}\beta }{\alpha }\left(-1-i{\text{sech}}\xi {\text{tanh}}\xi +{{\text{tanh}}}^{2}\xi \right),$$

58 $$V\left(\xi \right)=\frac{3\lambda \beta \dot{q}}{\alpha }\left(1+i{\text{sech}}\xi {\text{tanh}}\xi -{{\text{tanh}}}^{2}\xi \right).$$

Set 4: $${a}_{0}=-\frac{2{\lambda }^{2}\beta }{\alpha }$$, $${a}_{1}=0$$, $${a}_{2}=\frac{3{\lambda }^{2}\beta }{\alpha }$$, $${b}_{1}=0$$, $${b}_{2}=\pm \frac{3i{\lambda }^{2}\beta }{\alpha }$$, $$p=\lambda$$, $$q=\mu \pm {\lambda }^{2}\int \sqrt{\beta }dt$$.

Utilizing these findings, the method yields the solutions to the time-varying coefficient Boussinesq model as follows:

59 $$U\left(\xi \right)=\frac{{\lambda }^{2}\beta }{\alpha }\left(-2-3i{\text{sech}}\xi {\text{tanh}}\xi +3{{\text{tanh}}}^{2}\xi \right),$$

60 $$V\left(\xi \right)=\frac{\lambda \beta \dot{q}}{\alpha }\left(2+3i{\text{sech}}\xi {\text{tanh}}\xi -3{{\text{tanh}}}^{2}\xi \right).$$

In the above sets of solutions, $\xi =p\left(t\right)x+q(t)$.

## Results and discussion

In this section, we have discussed the exact results obtained by the three methods by considering various parameters through diagrams. We have depicted the profile of soliton solutions obtained by successfully applying the MSE method, the generalized Kudryashov method and the modified sine–Gordon technique to the Boussinesq system with varying coefficients. All the figures provide a remarkable representation of how the soliton solutions of an equation are affected by the variable coefficients of the equation. Selection of different types of functions for the variable coefficients in the system, soliton solutions exhibit a diverse range of waveforms or variations, which are displayed with 3D surfaces and 2D and 3D curves to highlight the variations. It is noticeable from the figures that the variable coefficients of the equation and the wave velocity function continuously change the shape of the wave surface, i.e. the length, height and direction of the wave. To highlight the authenticity of this phenomenon, we have diagrammed the wave velocity solutions (29)–(36), (43)–(50) and (53)–(60) that have physical applications in the real world.

### Graphical analysis of solutions: modified simple equation method

**Figure 1:**
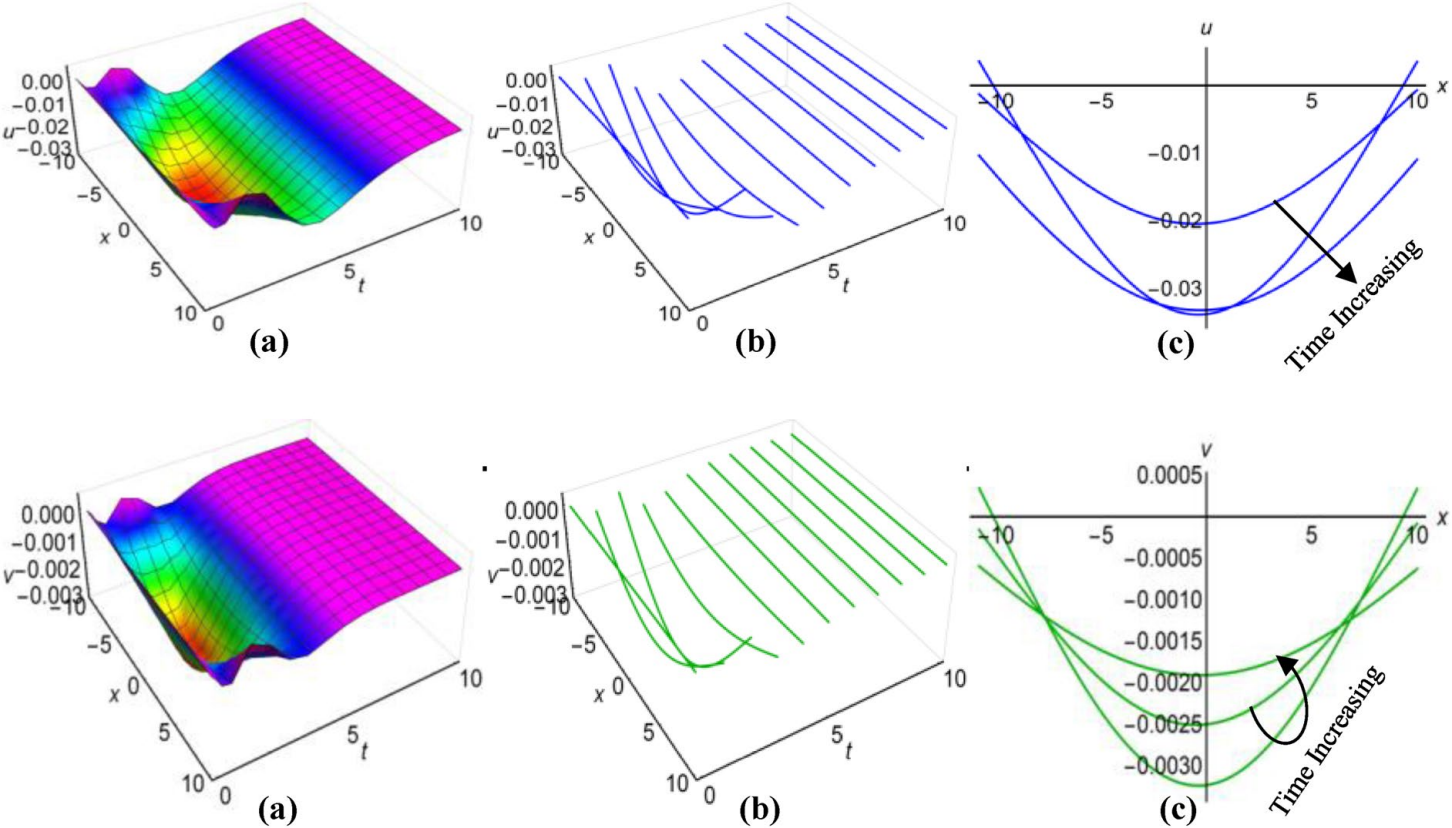
(i) Graphical representations of solution (29) for $u(x, t)$ when $q=\mu -\frac{1}{6}\int \frac{\alpha {a}_{1}}{\sqrt{\beta }}dt$. (**a**) Solution surface, (**b**) Solution curves in 3D form, (**c**) Solution curves in 2D form for $t=1, 2, 3$. (ii) Graphical representations of solution (30) for $v(x, t)$ when $q=\mu -\frac{1}{6}\int \frac{\alpha {a}_{1}}{\sqrt{\beta }}dt$. (**a**) Solution surface, (**b**) Solution curves in 3D form, (**c**) Solution curves in 2D form for $t=1, 2, 3$.

We have portrayed the results of the Boussinesq system with time-involving coefficients, marked as (29) and (30), for two distinct scenarios. In the first case, with $\lambda =1$, $\mu =0.6$, ${a}_{1}=t+1$, $\alpha ={e}^{-t}$, $\beta =\frac{1}{t}$ when $q=\mu -\frac{1}{6}\int \frac{\alpha {a}_{1}}{\sqrt{\beta }}dt$ and in the second case, with $\lambda =-0.65$, $\mu =0.2$, ${a}_{1}=2t$, $\alpha ={\text{cos}}3t$, $\beta =2{t}^{2}$ for $q=\mu +\frac{1}{6}\int \frac{\alpha {a}_{1}}{\sqrt{\beta }}dt$, we have presented these outcomes in Figs. 1(i), (ii) and 2 (i), (ii) respectively, spanning the intervals of $-10\le x\le 10$ and $0\le t\le 10$. Additionally, we have included both 2D and 3D solution curves, each associated with specific time value. The solution surfaces for both $u$ and $v$ form the anti-lump shaped waves in the first case, whereas the second case displays irregular periodic solitons with rising wave-amplitudes and wavelengths. On the other hand, the directions of the arrows in the (c) diagrams of all figures reveal how the 2D curves change with time.

**Figure 2:**
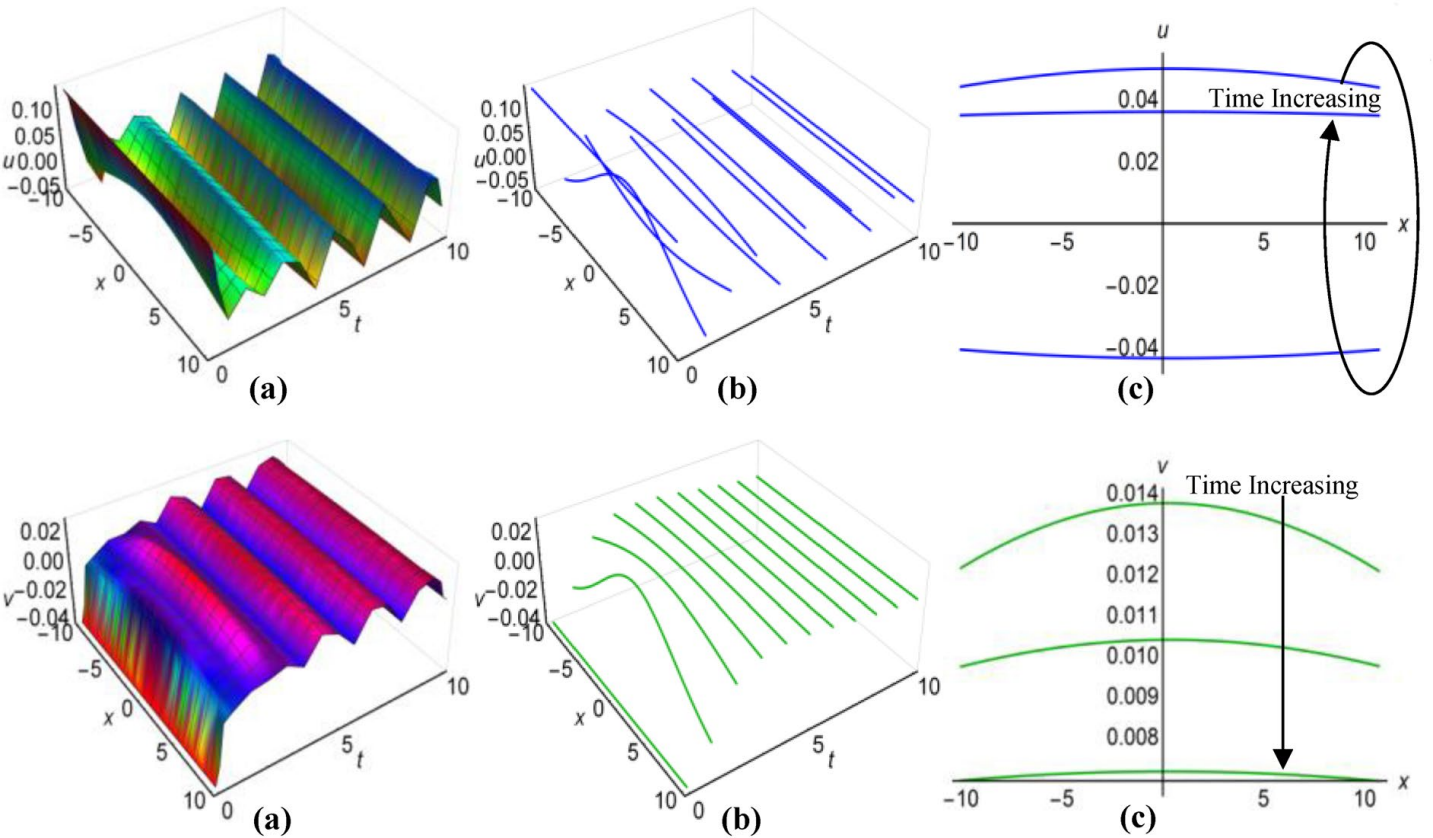
(i): Graphical representations of solution (29) for $u(x, t)$ when $q=\mu +\frac{1}{6}\int \frac{\alpha {a}_{1}}{\sqrt{\beta }}dt$. (**a**) Solution surface, (**b**) Solution curves in 3D form, (**c**) Solution curves in 2D form for $t=5, 6, 7$. (ii): Graphical representations of solution (30) for $v(x, t)$ when $q=\mu +\frac{1}{6}\int \frac{\alpha {a}_{1}}{\sqrt{\beta }}dt$. (**a**) Solution surface, (**b**) Solution curves in 3D form, (**c**) Solution curves in 2D form for $t=5, 6, 7$.

**Figure 3:**
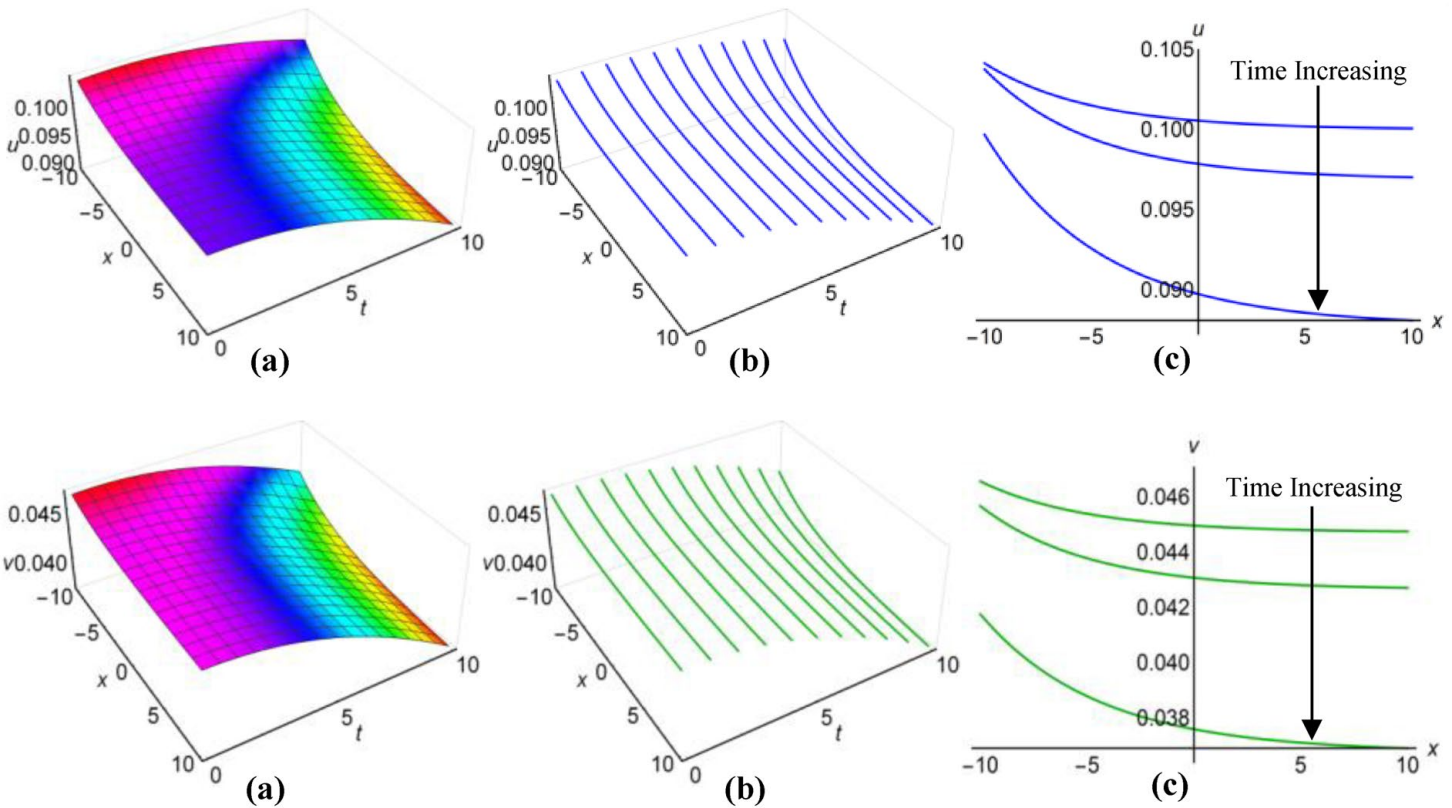
(i): Graphical representations of solution (31) for $u(x, t)$ when $q=\mu -\frac{1}{6}\int \frac{\alpha {a}_{1}}{\sqrt{\beta }}dt$.** a** Solution surface, **b** Solution curves in 3D form, **b** Solution curves in 2D form for *t* = 0, 5, 10. (ii): Graphical representations of solution (32) for $v(x, t)$ when $q=\mu -\frac{1}{6}\int \frac{\alpha {a}_{1}}{\sqrt{\beta }}dt$. **a** Solution surface, **b** Solution curves in 3D form, **c** Solution curves in 2D form for *t* = 0, 5, 10.

Furthermore, the results of the Boussinesq equations under changing coefficients, identified as (31) and (32), have represented graphically for $\lambda =-1$, $\mu =-26$, ${a}_{1}=3$, $\alpha =2$, $\beta =5{\text{sec}}\left(\frac{t}{20}\right)$, when $q=\mu -\frac{1}{6}\int \frac{\alpha {a}_{1}}{\sqrt{\beta }}dt$ and $\lambda =0.01$, $\mu =-0.5$, ${a}_{1}={\text{cosh}}t$, $\alpha =3$, $\beta =5{e}^{2t}$, when $q=\mu +\frac{1}{6}\int \frac{\alpha {a}_{1}}{\sqrt{\beta }}dt$, in Figs. 3(i), (ii) and 4(i), (ii) respectively, across the ranges $-10\le x\le 10$ and $0\le t\le 10$, including both 2D and 3D representations of solution curves at various time. The solution surfaces for the first and second cases represent curve-shaped and irregular-shaped solitons respectively.

**Figure 4:**
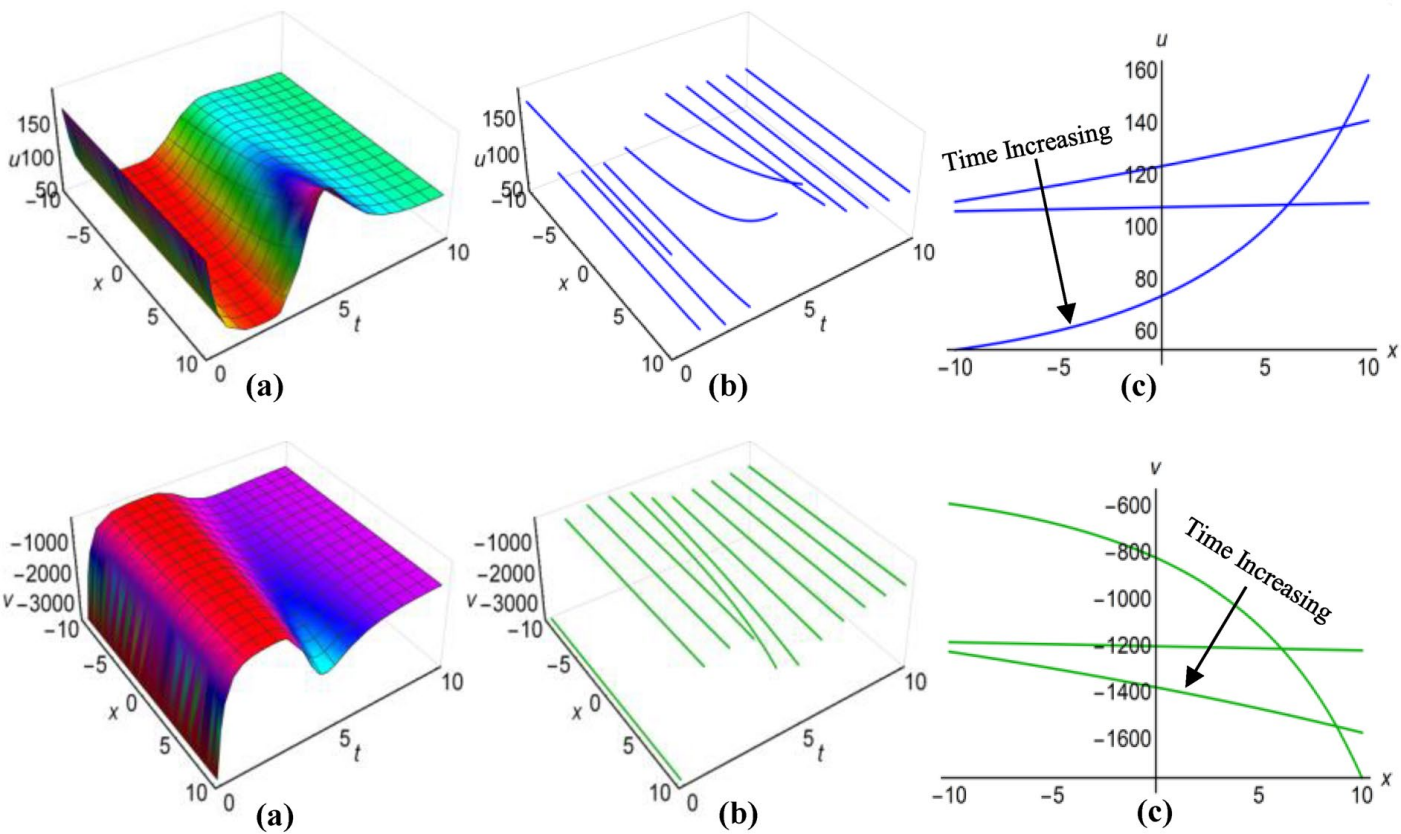
(i): Graphical representations of solution (31) for $u(x, t)$ when $q=\mu +\frac{1}{6}\int \frac{\alpha {a}_{1}}{\sqrt{\beta }}dt$. (**a**) Solution surface, (**b**) Solution curves in 3D form, (**c**) Solution curves in 2D form for *t* = 4, 6, 8. (ii): Graphical representations of solution (32) for $v(x, t)$ when $q=\mu +\frac{1}{6}\int \frac{\alpha {a}_{1}}{\sqrt{\beta }}dt$. (**a**) Solution surface, (**b**) Solution curves in 3D form, (**c**) Solution curves in 2D form for *t* = 4, 6, 8.

**Figure 5:**
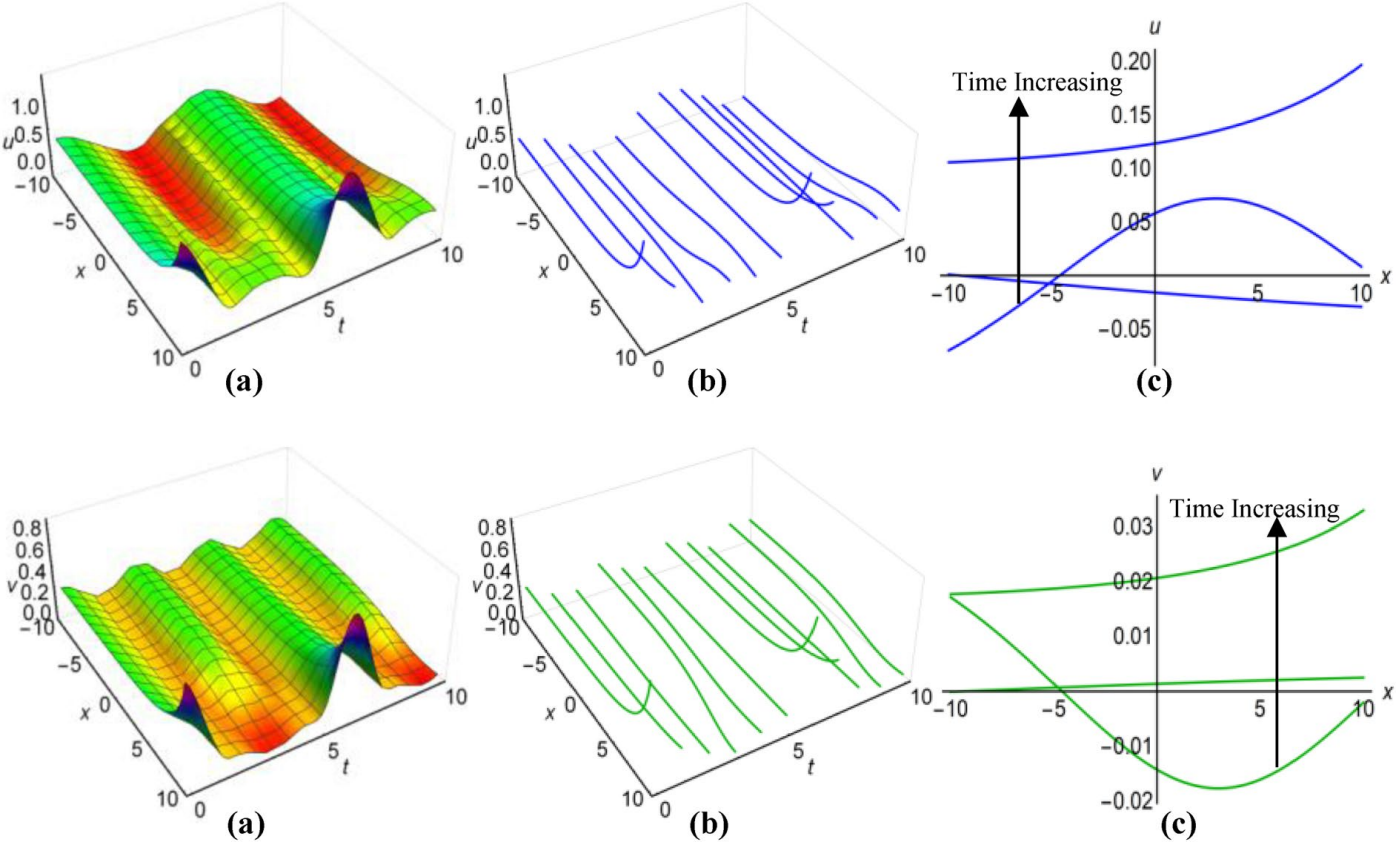
(i): Graphical representations of solution (33) for $u(x, t)$ when $q=\mu -\frac{1}{6}\int \frac{\alpha {a}_{1}}{\sqrt{\beta }}dt$. **a**) Solution surface, (**b**) Solution curves in 3D form, (**c**) Solution curves in 2D form for $t=2, 5, 8$. (ii): Graphical representations of solution (34) for $v(x, t)$ when $q=\mu -\frac{1}{6}\int \frac{\alpha {a}_{1}}{\sqrt{\beta }}dt$. (**a**) Solution surface, (**b**) Solution curves in 3D form, (**c**) Solution curves in 2D form for $t=2, 5, 8$.

Again for $\lambda =0.1$, $\mu =-1.15$, ${a}_{1}=0.5$, $\alpha ={\text{cos}}t$, $\beta =2$, $q=\mu -\frac{1}{6}\int \frac{\alpha {a}_{1}}{\sqrt{\beta }}dt$ and $\lambda =0.1$, $\mu =-0.6$, ${a}_{1}=3{\text{tanh}}t$, $\alpha =1$, $\beta =2{\text{sinh}}t$, $q=\mu +\frac{1}{6}\int \frac{\alpha {a}_{1}}{\sqrt{\beta }}dt$, we have plotted the solutions of the time-varying coefficient Boussinesq model, denoted as (33) and (34), on Figs. 5(i), (ii) and 6(i), (ii) respectively. These plots encompassed the spatial domain $-10\le x\le 10$ and the temporal domain $0\le t\le 10$, also presented solution curves for distinct time points in both 2D and 3D diagrams. In the first scenario, the solution surfaces represent irregular periodic solitons, while they depict bell-shaped waves in the second scenario.

**Figure 6:**
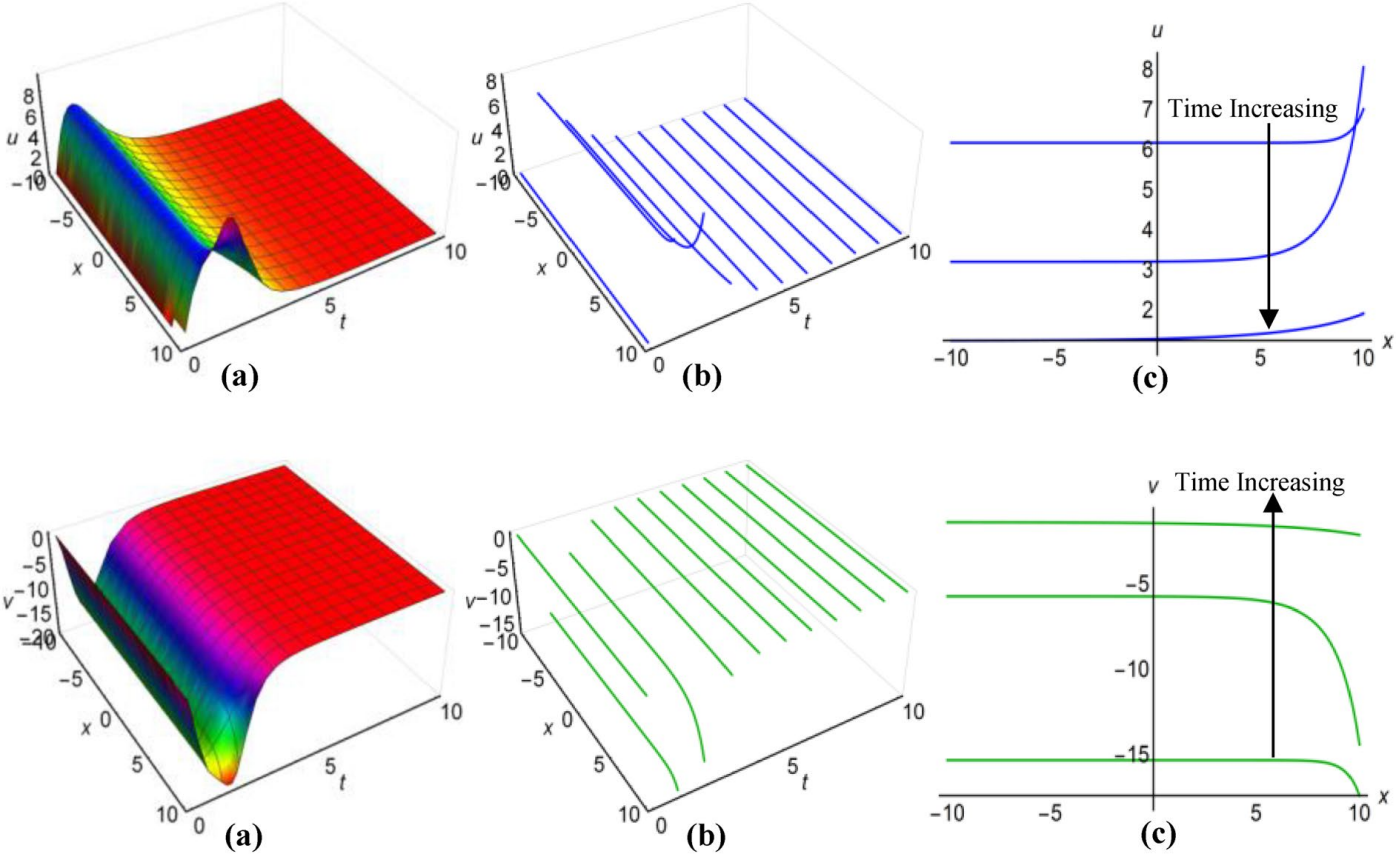
(i): Graphical representations of solution (33) for $u(x, t)$ when $q=\mu +\frac{1}{6}\int \frac{\alpha {a}_{1}}{\sqrt{\beta }}dt$. (**a**) Solution surface, (**b**) Solution curves in 3D form, (**c**) Solution curves in 2D form for $t=1, 2, 3$. (ii): Graphical representations of solution (34) for $v(x, t)$ when $q=\mu +\frac{1}{6}\int \frac{\alpha {a}_{1}}{\sqrt{\beta }}dt$. (**a**) Solution surface, (**b**) Solution curves in 3D form, (**c**) Solution curves in 2D form for $t=1, 2, 3$.

### Graphical analysis of solutions: generalized Kudryashov method

**Figure 7:**
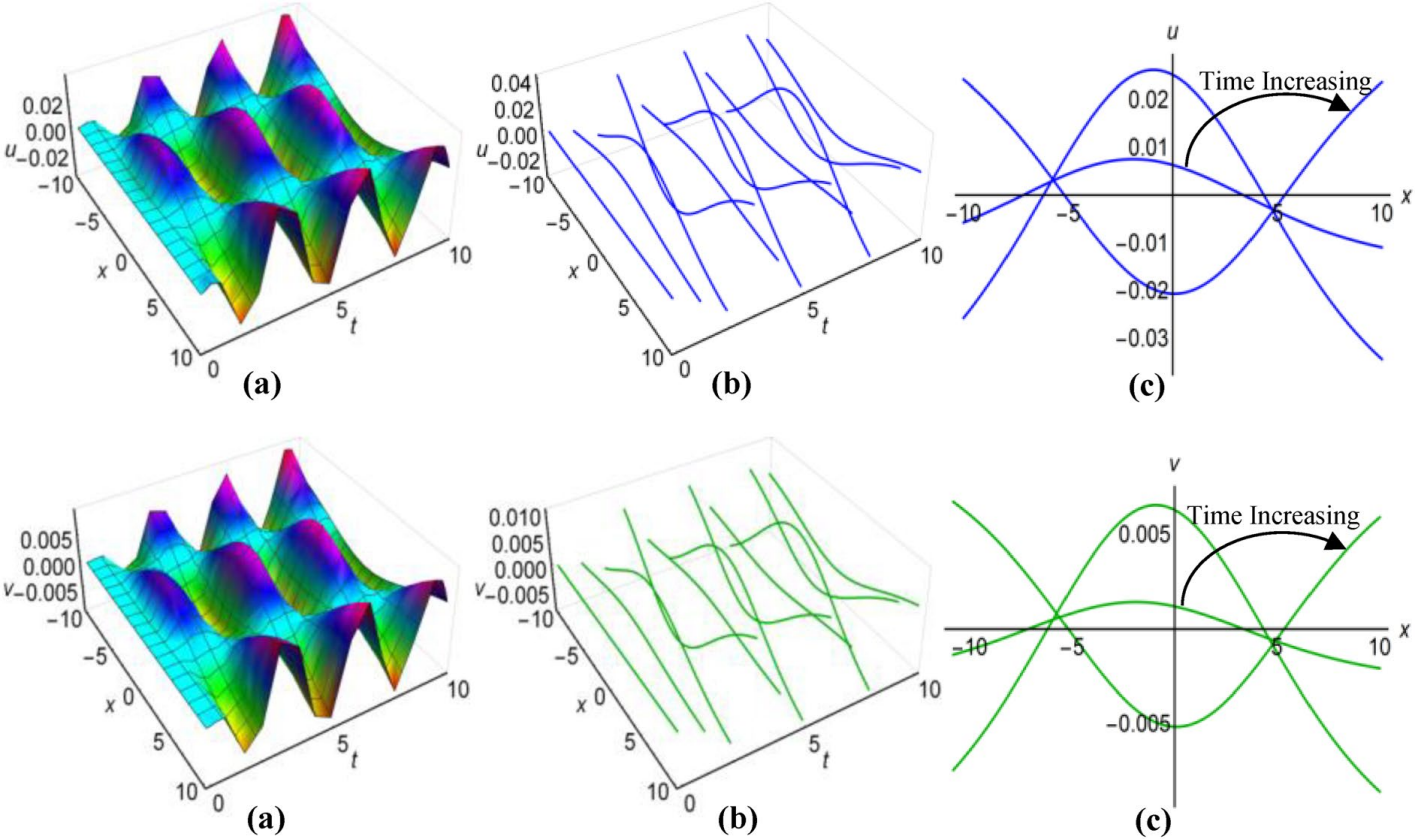
(i): Graphical representations of solution (43) for $u(x, t)$ when $q=\mu -{\uplambda }^{2}\int \sqrt{\beta }dt$. (**a**) Solution surface, (**b**) Solution curves in 3D form, (**c**) Solution curves in 2D form for $t=1, 5, 9$. (ii): Graphical representations of solution (44) for $v(x, t)$ when $q=\mu -{\uplambda }^{2}\int \sqrt{\beta }dt$. (**a**) Solution surface, (**b**) Solution curves in 3D form, (**c**) Solution curves in 2D form for $t=1, 5, 9$.

For $\lambda =0.25$, $\mu =0.5$, $\alpha ={\text{sec}}2t$, $\beta ={{\text{tanh}}}^{2}t$ when $q=\mu -{\uplambda }^{2}\int \sqrt{\beta }dt$ and $\lambda =1$, $\mu =-15$, $\alpha =10(t+1)$, $\beta =\frac{{\left({t}^{2}-2t+2\right)}^{2}}{15}$ when $q=\mu +{\uplambda }^{2}\int \sqrt{\beta }dt$, the two results (43) and (44) of the Boussinesq system characterized by time-varying coefficients, have depicted in Figs. 7(i), (ii) and 8(i), (ii) over the limitations $-10\le x\le 10$ and $0\le t\le 10$, together with 2D and 3D representations of solution curves across different time. In the both cases, the solution surfaces take on distinct forms, with the first case showing double-periodic solitons in both directions and the second case revealing irregular half-range kink with anti-bell and half-range anti-kink with bell shaped solitons along $t$-axis for $u(x, t)$ and $v(x, t)$ respectively.

**Figure 8:**
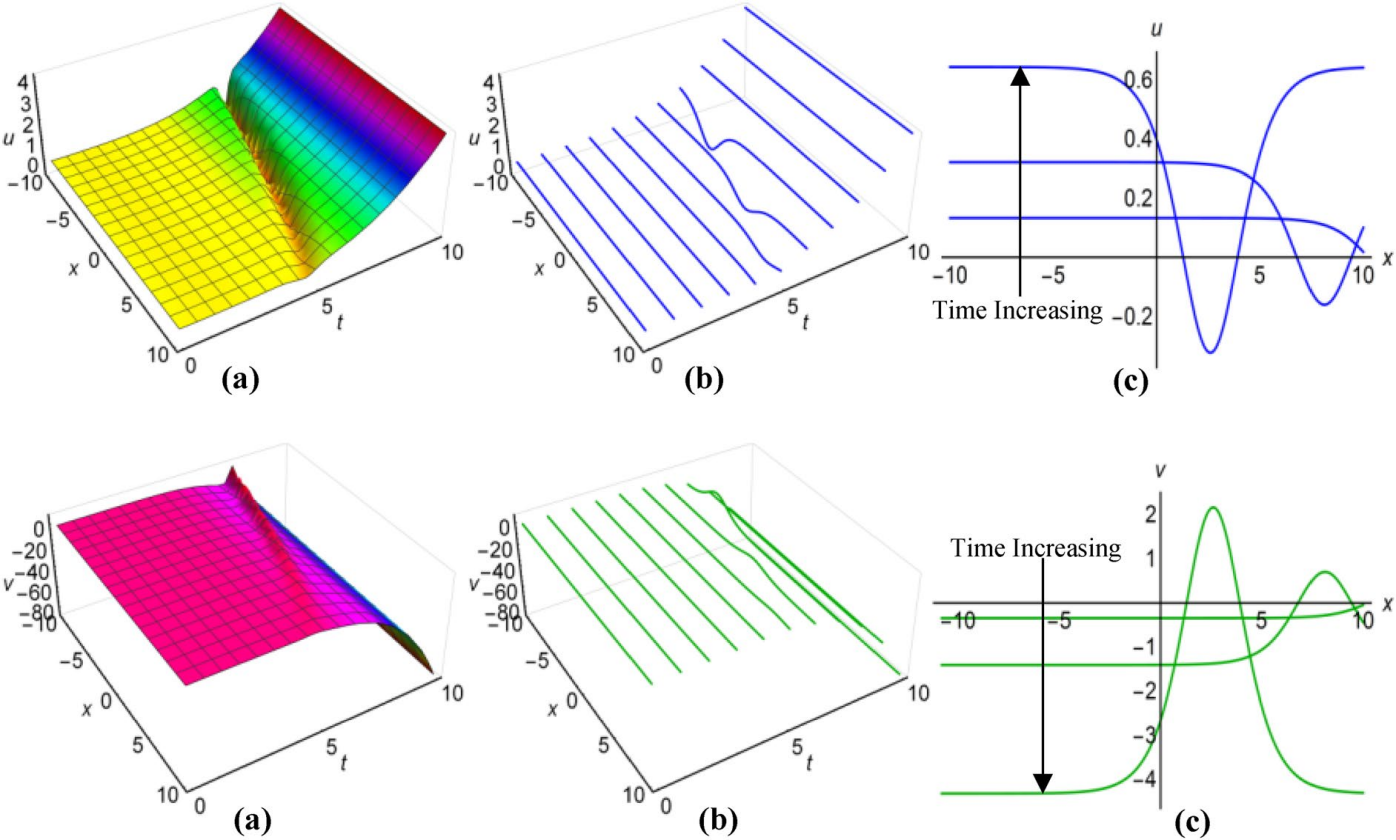
(i): Graphical representations of solution (43) for $u(x, t)$ when $q=\mu +{\uplambda }^{2}\int \sqrt{\beta }dt$. (**a**) Solution surface, (**b**) Solution curves in 3D form, (**c**) Solution curves in 2D form for $t=4, 5, 6$. (ii): Graphical representations of solution (44) for $v(x, t)$ when $q=\mu +{\uplambda }^{2}\int \sqrt{\beta }dt$. (**a**) Solution surface, (**b**) Solution curves in 3D form, (**c**) Solution curves in 2D form for $t=4, 5, 6$.

**Figure 9:**
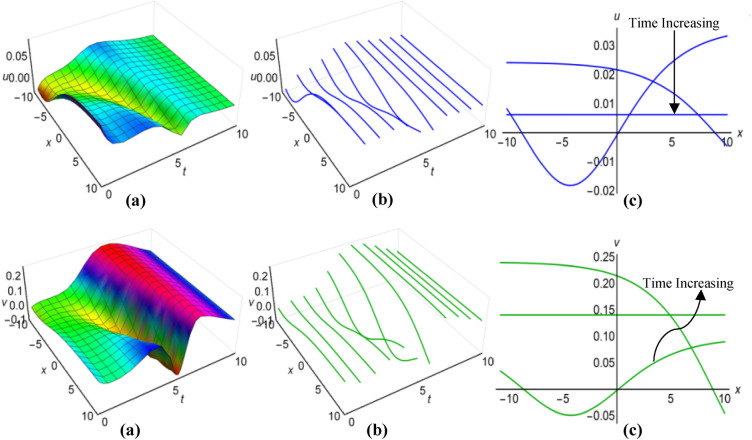
(i): Graphical representations of solution (45) for $u(x, t)$ when $q=\mu -{\uplambda }^{2}\int \sqrt{\beta }dt$. (**a**) Solution surface, (**b**) Solution curves in 3D form, (**c**) Solution curves in 2D form for $t=3, 6, 9$. (ii): Graphical representations of solution (46) for $v(x, t)$ when $q=\mu -{\uplambda }^{2}\int \sqrt{\beta }dt$. (**a**) Solution surface, (**b**) Solution curves in 3D form, (**c**) Solution curves in 2D form for $t=3, 6, 9$.

Within the Boussinesq equations with time-evolving coefficients, we have displayed the two results identified as (45) and (46) in Figs. 9(i), (ii) and 10(i), (ii) throughout the region where $-10\le x\le 10$ and $0\le t\le 10$ for two specific conditions. In the first condition, with $\lambda =0.3$, $\mu =1.8$, $\alpha =10{e}^{t}$, $\beta ={({t}^{2}-t+3)}^{2}$ in situation where $q=\mu -{\uplambda }^{2}\int \sqrt{\beta }dt$ and in the second condition, featuring $\lambda =-0.35$, $\mu =0.95$, $\alpha ={\text{sec}}{e}^{t/5}$, $\beta ={0.5}^{{\text{t}}/5}$ whenever $q=\mu +{\uplambda }^{2}\int \sqrt{\beta }dt$ have considered. Additionally these figures show both 2D and 3D solution curves for different values of time. Irregular anti-bell shaped solitons characterize the solution surfaces in the first case, whereas the second case displays irregular double-periodic solitons with respect to both axes.

**Figure 10:**
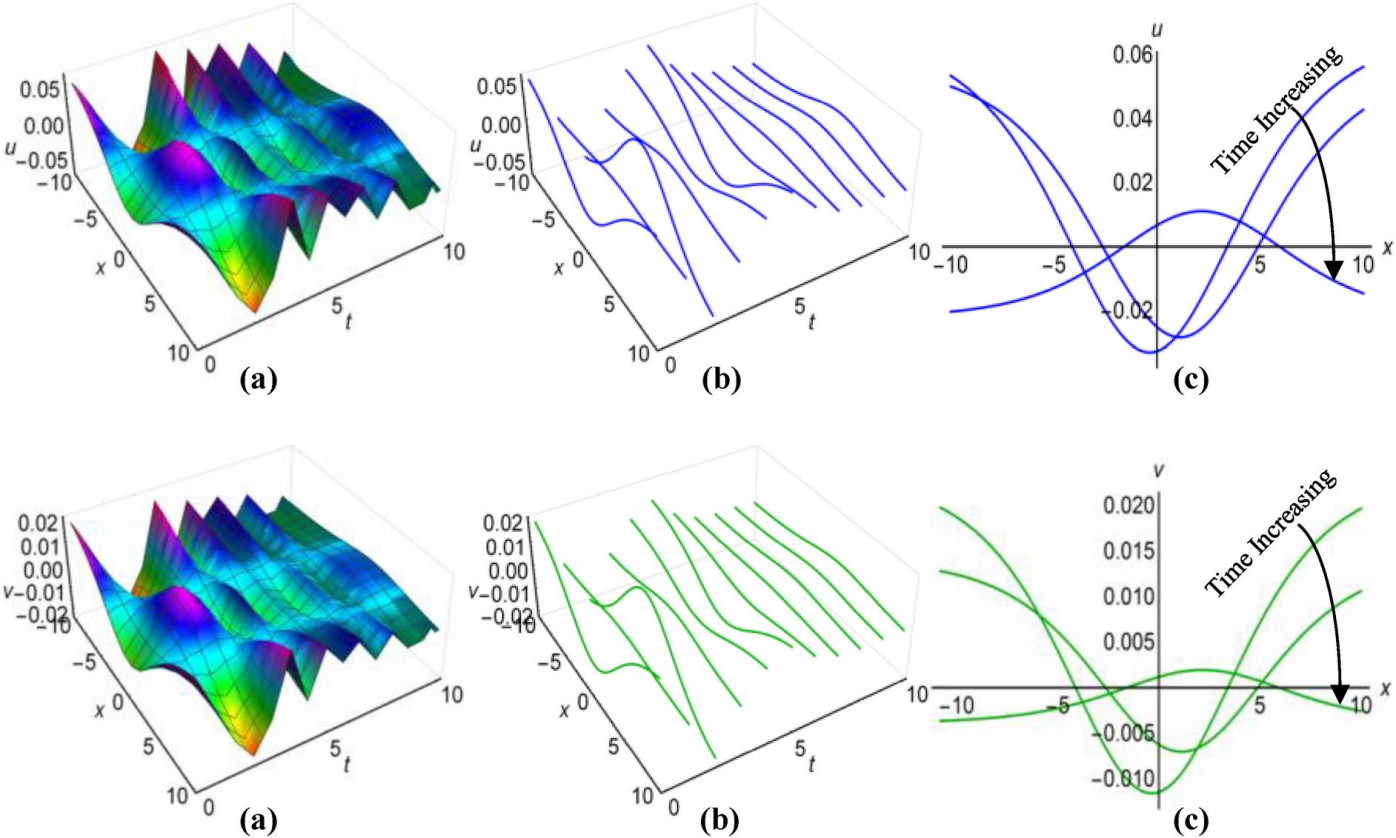
(i): Graphical representations of solution (45) for $u(x, t)$ when $q=\mu +{\uplambda }^{2}\int \sqrt{\beta }dt$. (**a**) Solution surface, (**b**) Solution curves in 3D form, (**c**) Solution curves in 2D form for $t=0, 5, 10$. (ii): Graphical representations of solution (46) for $v(x, t)$ when $q=\mu +{\uplambda }^{2}\int \sqrt{\beta }dt$. (**a**) Solution surface, (**b**) Solution curves in 3D form, (**c**) Solution curves in 2D form for $t=0, 5, 10$.

**Figure 11:**
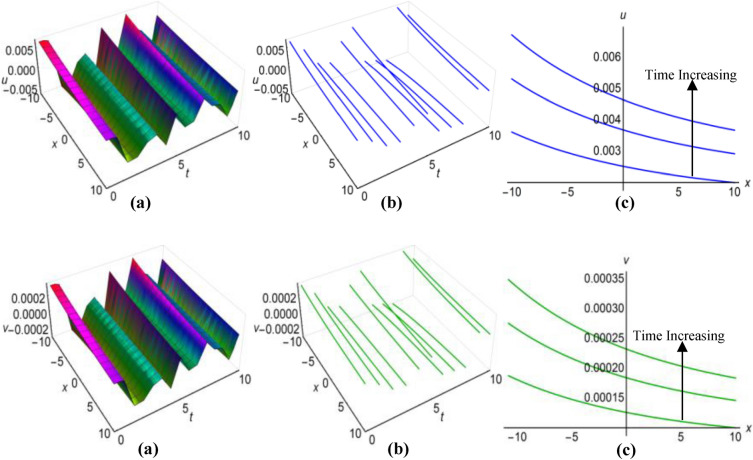
(i): Graphical representations of solution (47) for $u(x, t)$ when $q=\mu -{\uplambda }^{2}\int \sqrt{\beta }dt$. (**a**) Solution surface, (**b**) Solution curves in 3D form, (**c**) Solution curves in 2D form for $t=1, 5, 9$. (ii): Graphical representations of solution (48) for $v(x, t)$ when $q=\mu -{\uplambda }^{2}\int \sqrt{\beta }dt$. (**a**) Solution surface, (**b**) Solution curves in 3D form, (**c**) Solution curves in 2D form for $t=1, 5, 9$.

In the case of $\lambda =0.05$, $\mu =1.5$, $\alpha ={\text{sec}}{t}^{3}$, $\beta =1$ when $q=\mu -{\uplambda }^{2}\int \sqrt{\beta }dt$ and for $\lambda =-0.16$, $\mu =4.85$, $\alpha =10{\text{csc}}{e}^{3t}$, $\beta ={{\text{cos}}}^{2}\frac{t}{8}$ with $q=\mu +{\uplambda }^{2}\int \sqrt{\beta }dt$, we have displayed the two results (47) and (48) of the Boussinesq system with time-influenced coefficients in Figs. 11(i), (ii) and 12(i), (ii) within the boundaries of $-10\le x\le 10$ and $0\le t\le 10$, along with 2D and 3D representations of solution curves at different points of time. In both cases the two solution surfaces show irregular periodic solitons, but they exhibit their distinct properties.

**Figure 12:**
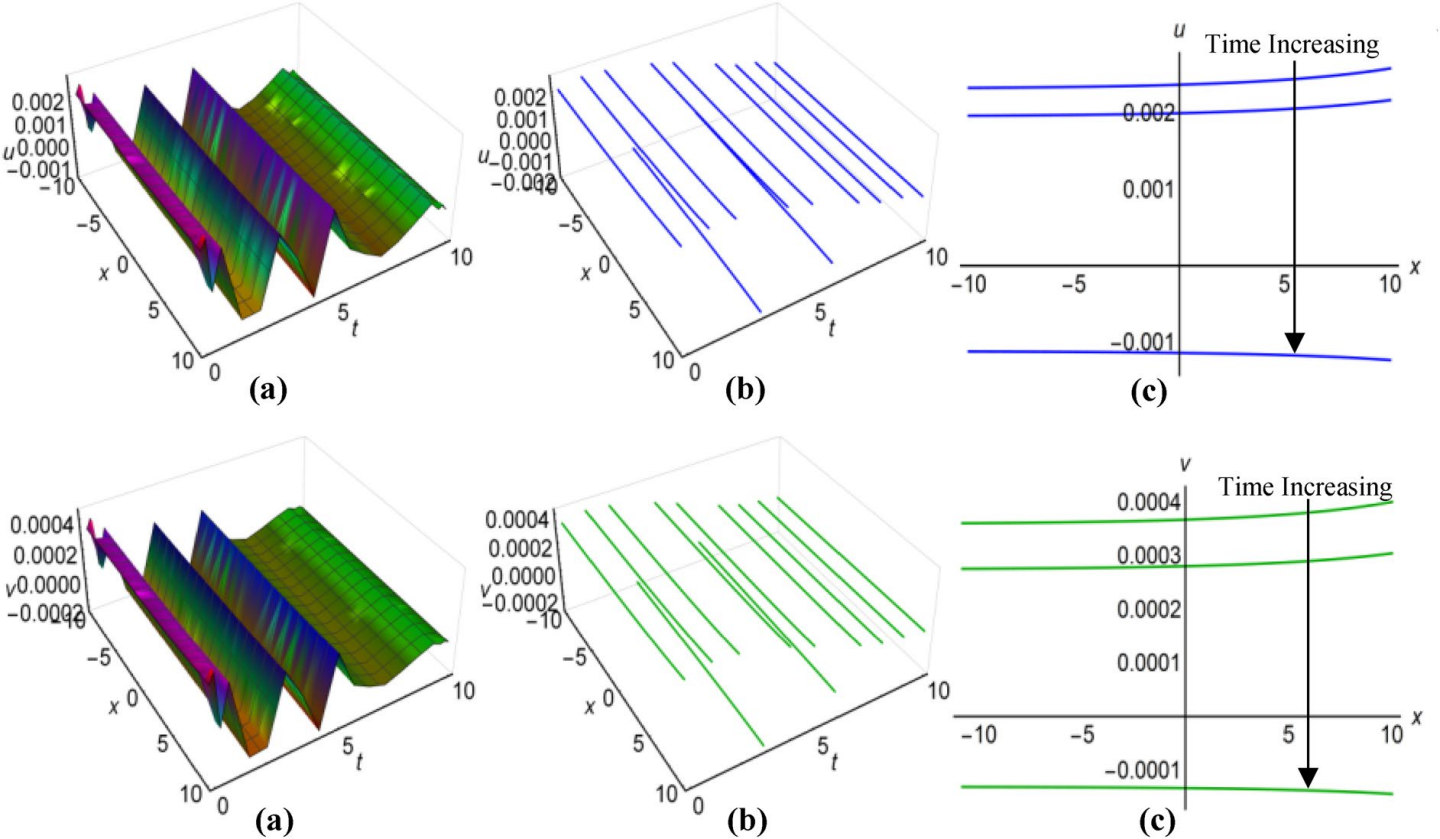
(i): Graphical representations of solution (47) for $u(x, t)$ when $q=\mu +{\uplambda }^{2}\int \sqrt{\beta }dt$. (**a**) Solution surface, (**b**) Solution curves in 3D form, (**c**) Solution curves in 2D form for $t=2, 4, 6$. (ii): Graphical representations of solution (48) for $v(x, t)$ when $q=\mu +{\uplambda }^{2}\int \sqrt{\beta }dt$. (**a**) Solution surface, (**b**) Solution curves in 3D form, (**c**) Solution curves in 2D form for $t=2, 4, 6$.

### Graphical analysis of solutions: modified sine–Gordon expansion method

**Figure 13:**
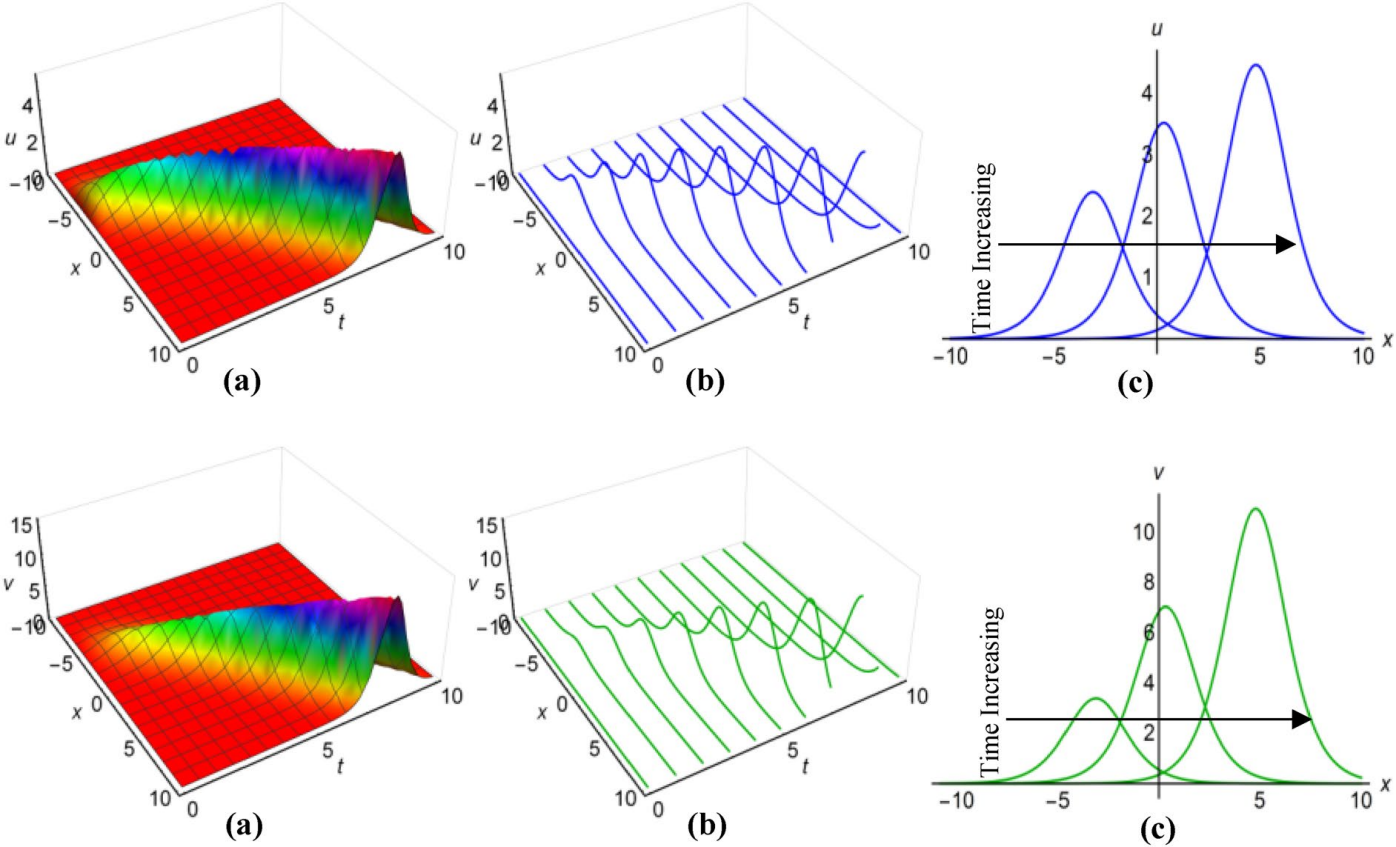
(i): Graphical representations of solution (53) for $u(x, t)$ when $q=\mu -2i{\lambda }^{2}\int \sqrt{\beta }dt$. (**a**) Solution surface, (**b**) Solution curves in 3D form, (**c**) Solution curves in 2D form for $t=2, 4, 6$. (ii): Graphical representations of solution (54) for $v(x, t)$ when $q=\mu -2i{\lambda }^{2}\int \sqrt{\beta }dt$. (**a**) Solution surface, (**b**) Solution curves in 3D form, (**c**) Solution curves in 2D form for $t=2, 4, 6$.

**Figure 14:**
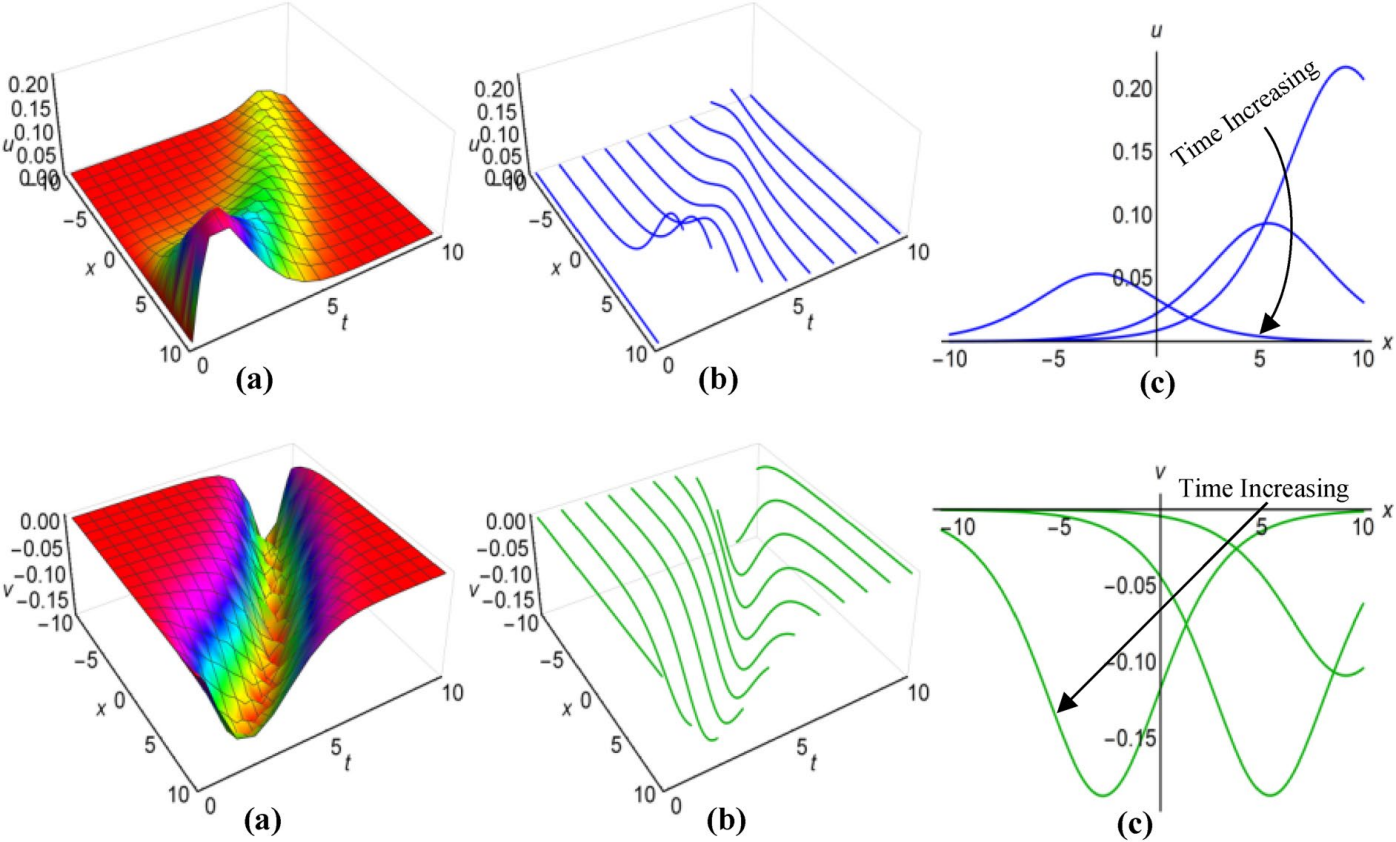
(i): Graphical representations of solution (53) for $u(x, t)$ when $q=\mu +2i{\lambda }^{2}\int \sqrt{\beta }dt$. (**a**) Solution surface, (**b**) Solution curves in 3D form, (**c**) Solution curves in 2D form for $t=1, 4, 7$. (ii): Graphical representations of solution (54) for $v(x, t)$ when $q=\mu +2i{\lambda }^{2}\int \sqrt{\beta }dt$. (**a**) Solution surface, (**b**) Solution curves in 3D form, (**c**) Solution curves in 2D form for $t=1, 4, 7$.

In Fig. 13(i) and (ii), as well as Fig. 14(i) and (ii), we have illustrated the solutions to the Boussinesq model, identified as (53) and (54) for the two sets of parameters $\lambda =0.5$, $\mu =2.5$, $\alpha ={\text{ln}}(t+1.5)$, $\beta =-t$, where $q=\mu -2i{\lambda }^{2}\int \sqrt{\beta }dt$ and $\lambda =0.25$, $\mu =-2.35$, $\alpha ={t}^{3}{{\text{coth}}}^{2}t$, $\beta =-{t}^{2}$, when $q=\mu +2i{\lambda }^{2}\int \sqrt{\beta }dt$. These graphical representations span the area $-10\le x\le 10$ and $0\le t\le 10$, also incorporate the representations of both solution curves for various time points in 2D and 3D diagrams. In both cases, solitons with bell-shaped and anti-bell-shaped profiles, subject to parabolic tapering, represent the respective solution surfaces.

**Figure 15:**
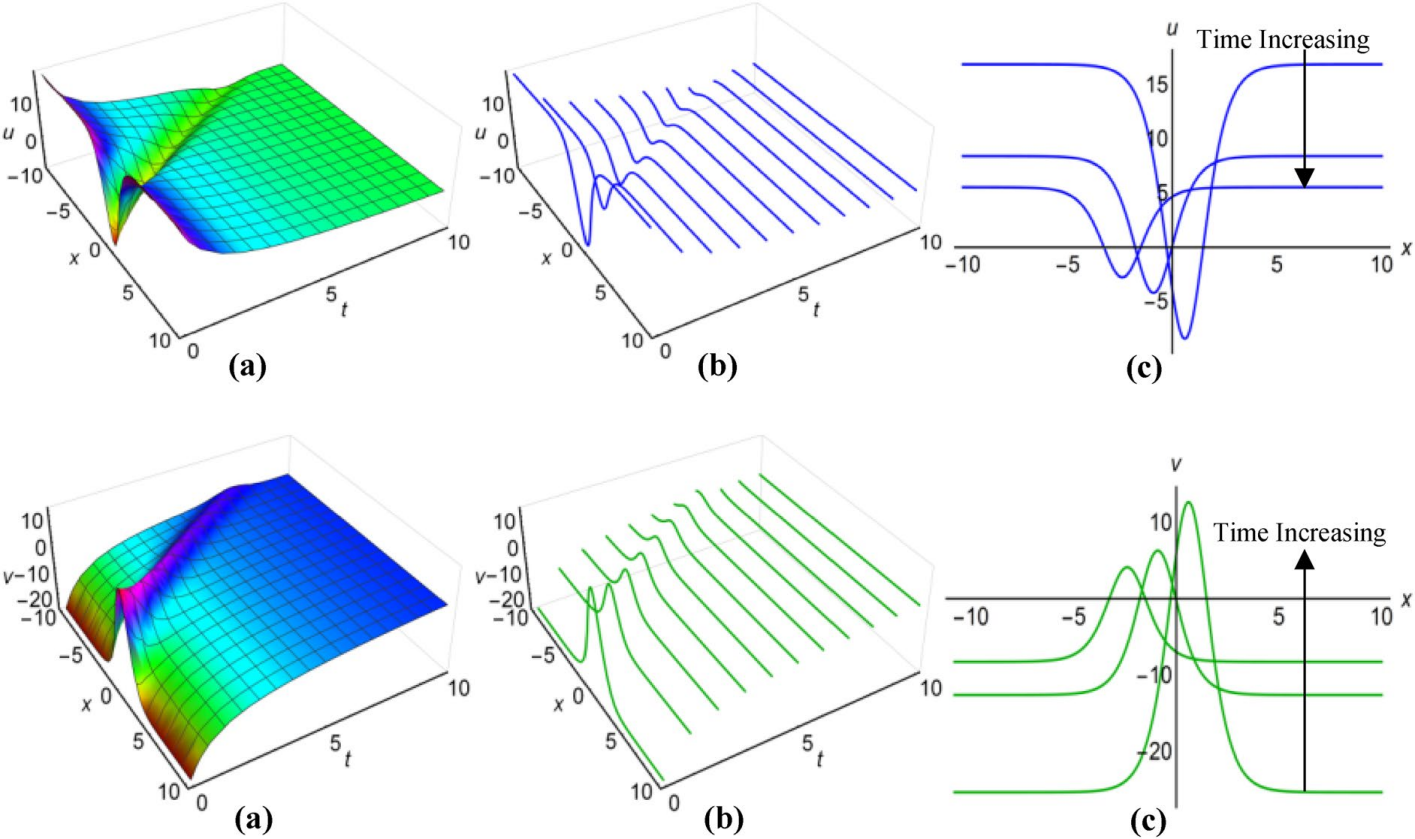
(i): Graphical representations of solution (55) for $u(x, t)$ when $q=\mu -2{\lambda }^{2}\int \sqrt{\beta }dt$. (**a**) Solution surface, (**b**) Solution curves in 3D form, (**c**) Solution curves in 2D form for $t=0, 1, 2$. (ii): Graphical representations of solution (56) for $v(x, t)$ when $q=\mu -2{\lambda }^{2}\int \sqrt{\beta }dt$. (**a**) Solution surface, (**b**) Solution curves in 3D form, (**c**) Solution curves in 2D form for $t=0, 1, 2$.

Using $\lambda =-0.75$, $\mu =0.45$, $\alpha =2{\text{sin}}\left(\frac{t+1}{15}\right)$, $\beta ={\text{cos}}\left(\frac{t}{10}\right)$, for $q=\mu -2{\lambda }^{2}\int \sqrt{\beta }dt$ and $\lambda =0.15$, $\mu =-0.35$, $\alpha =3{\text{sec}}t$, $\beta ={\text{ln}}(t+3)$, for $q=\mu +2{\lambda }^{2}\int \sqrt{\beta }dt$, we have generated graphical representations in Figs. 15(i), (ii) and 16(i), (ii) for the results (55) and (56) of the Boussinesq model, covering the bounded field where $-10\le x\le 10$ and $0\le t\le 10$, with additional 2D and 3D explanations of solution curves reflecting diverse time increments. For the first case, both solution surfaces portray anti-bell and bell-shaped solitons with parabolic tapering; and in the second case, they delineate double-periodic solitons for both $u(x, t)$ and $v(x, t)$, respectively.

**Figure 16:**
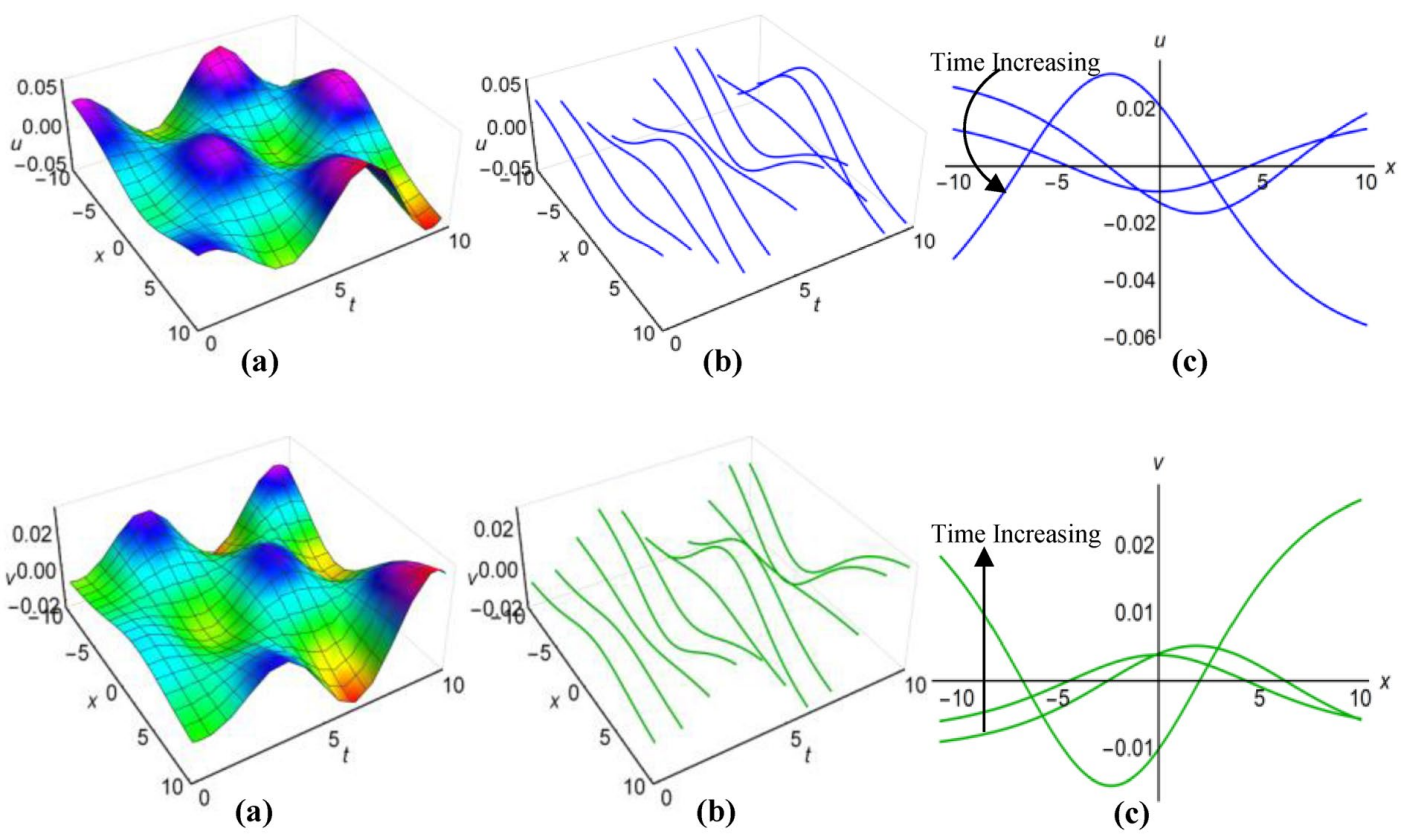
(i): Graphical representations of solution (55) for $u(x, t)$ when $q=\mu +2{\lambda }^{2}\int \sqrt{\beta }dt$. (ii): Graphical representations of solution (56) for $v(x, t)$ when $q=\mu +2{\lambda }^{2}\int \sqrt{\beta }dt$. (**a**) Solution surface, (**b**) Solution curves in 3D form, (**c**) Solution curves in 2D form for $t=0, 5, 10$.

**Figure 17:**
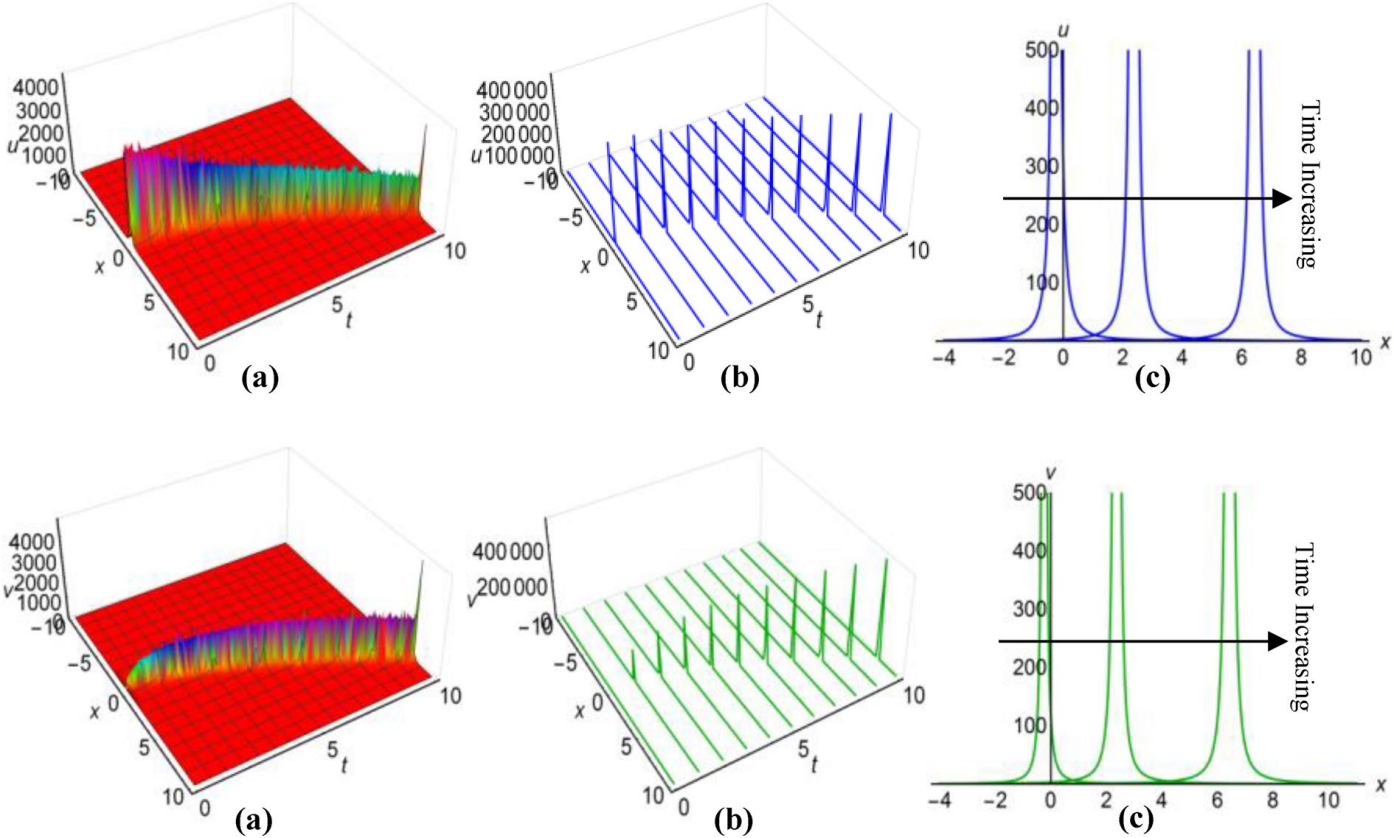
(i): Graphical representations of solution (35) for $u(x, t)$ when $q=\mu -\frac{1}{6}\int \frac{\alpha {a}_{1}}{\sqrt{\beta }}dt$. (**a**) Solution surface, (**b**) Solution curves in 3D form, (**c**) Solution curves in 2D form for $t=1, 5, 9$. (ii): Graphical representations of solution (36) for $v(x, t)$ when $q=\mu -\frac{1}{6}\int \frac{\alpha {a}_{1}}{\sqrt{\beta }}dt$. (**a**) Solution surface, (**b**) Solution curves in 3D form, (**c**) Solution curves in 2D form for $t=1, 5, 9$.

Finally, we have refrained from showing figures for singular solutions (35), (36), (49), (50), (57)-(60) of the Boussinesq model, which are mathematically correct but have no physical application in the real world. Although sometimes considering special functions for variable coefficients, some of these solutions behave like non-singular solutions. Thus we have shown only the diagrams of solutions (35) and (36) in Fig. 17(i) and (ii) rather than illustrating all the graphs of those solutions. We have considered $\lambda =1$, $\mu =0.5$, ${a}_{1}=4$, $\alpha =t$, $\beta =3t$ and taking $q=\mu -\frac{1}{6}\int \frac{\alpha {a}_{1}}{\sqrt{\beta }}dt$ to display the figures for $-10\le x\le 10$ and $0\le t\le 10$, where both images reveal singular periodic solitons. Also in these figures the solution curves for different values of time are presented in 2D and 3D diagrams.

From the above discussion it is clear that the applied methods serve as suitable, applicable and very effective tools to find the soliton solutions of the nonlinear evolution models with variable coefficients, which are developed in engineering, physics and applied sciences.

## Conclusion

In this study, we have developed substantial techniques for obtaining exact solutions to the Boussinesq systems characterized by the time-varying coefficients. The accessibility of these solutions through the utilization of the modified simple equation, the modified sine–Gordon expansion and the Kudryashov methods open up new paths for understanding and modeling intricate systems with variable coefficients. The explanations and visual presentations of the attained results for different cases increase the accessibility of our findings and enable a more intuitive grasp of the dynamics of the system. This research provides theoretical understanding as well as opportunities for advanced problem-solving technique by bridging the gap between constant and variable coefficient situations in Boussinesq equations. Although these methods have advantages, they encounter limitations when applied to solving equations. These limitations include sensitivity to changes in coefficients, challenges in presenting a physically meaningful interpretation, and the generation of impractical solutions. To address these constraints, there is a need to refine existing methods and explore hybrid approaches. This study might be used both in theory and in practice, which will make a significant contribution to thorough research in mathematical physics, which will act as a catalyst in the investigation of other problems in the future.

## Data Availability

No data was used for the research described in the article.

## References

[1] Ismael HF, Akkilic AN, Murad MAS, Bulut H, Mahmoud W, Osman MS (2022). Boiti-Leon-Manna-Pempinelli equation including time-dependent coefficient (vcBLMPE): A variety of nonautonomous geometrical structures of wave solutions. Nonlinear Dyn..

[2] Khater MM (2023). Characterizing shallow water waves in channels with variable width and depth; computational and numerical simulations. Chaos Solitons Fract..

[3] Ahmed GS (2012). A numerical algorithm for solving advection-diffusion equation with constant and variable coefficients. Open Numer. Methods J..

[4] Pinar Z (2020). Analytical studies for the Boiti–Leon–Monna–Pempinelli equations with variable and constant coefficients. Asymptot. Anal..

[5] Kabir MM (2011). Modified Kudryashov method for generalized forms of the nonlinear heat conduction equation. Int. J. Phys. Sci.

[6] Rizvi STR, Seadawy AR, Ali K, Ashraf MA, Althubiti S (2022). Multiple lump and interaction solutions for fifth-order variable coefficient nonlinear-Schrödinger dynamical equation. Opt. Quant. Electron..

[7] Gao XY, Guo YJ, Shan WR (2022). Similarity reductions for a generalized (3+1)-dimensional variable-coefficient B-type Kadomtsev–Petviashvili equation in fluid dynamics. Chin. J. Phys..

[8] Khatun MM, Akbar MA (2023). Analytical soliton solutions of the beta time-fractional simplified modified Camassa-Holm equation in shallow water wave propagation. J. Umm Al-Qura Univ. Appl. Sci..

[9] Arnous AH, Biswas A, Asma M, Belic M (2018). Dark and singular solitons in optical metamaterials with anti-cubic nonlinearity by modified simple equation approach. Optoelectron. Adv. Mater.-Rapid Commun..

[10] Yang Y, Gao YX, Yang HW (2021). Analysis of the rogue waves in the blood based on the high-order NLS equations with variable coefficients. Chin. Phys. B.

[11] Anwar N, Ahmad I, Kiani AK, Shoaib M, Raja MAZ (2023). Intelligent solution predictive networks for non-linear tumor-immune delayed model. Comput. Methods Biomech. Biomed. Eng..

[12] Sabir Z, Akkurt N, Said SB (2023). A novel radial basis Bayesian regularization deep neural network for the Maxwell nanofluid applied on the Buongiorno model. Arab. J. Chem..

[13] Ayub A, Sabir Z, Shah SZH, Mahmoud SR, Algarni A, Sadat R, Ali MR (2022). Aspects of infinite shear rate viscosity and heat transport of magnetized Carreau nanofluid. Eur. Phys. J. Plus.

[14] Shoaib M, Anwar N, Ahmad I, Naz S, Kiani AK, Raja MAZ (2023). Neuro-computational intelligence for numerical treatment of multiple delays SEIR model of worms propagation in wireless sensor networks. Biomed. Signal Process. Control.

[15] Anwar N, Ahmad I, Fatima A, Kiani AK, Shoaib M, Raja MAZ (2023). Design of intelligent Bayesian supervised predictive networks for nonlinear delay differential systems of avian influenza model. Eur. Phys. J. Plus.

[16] Anwar N, Ahmad I, Kiani AK, Shoaib M, Raja MAZ (2023). Novel intelligent Bayesian computing networks for predictive solutions of nonlinear multi-delayed tumor oncolytic virotherapy systems. Int. J. Biomath..

[17] Souayeh B, Sabir Z (2023). Designing hyperbolic tangent sigmoid function for solving the Williamson nanofluid model. Fract. Fract..

[18] Anwar N, Shoaib M, Ahmad I, Naz S, Kiani AK, Raja MAZ (2023). Intelligent computing networks for nonlinear influenza-A epidemic model. Int. J. Biomath..

[19] Botmart T, Sabir Z, Raja MAZ, Sadat R, Ali MR (2023). Stochastic procedures to solve the nonlinear mass and heat transfer model of Williamson nanofluid past over a stretching sheet. Ann. Nucl. Energy.

[20] Wahab HA, Shah SZH, Ayub A, Sabir Z, Sadat R, Ali MR (2023). Heterogeneous/homogeneous and inclined magnetic aspect of infinite shear rate viscosity model of Carreau fluid with nanoscale heat transport. Arab. J. Chem..

[21] Sabir Z, Imran A, Umar M, Zeb M, Shoaib M, Raja MAZ (2021). A numerical approach for 2-D Sutterby fluid-flow bounded at a stagnation point with an inclined magnetic field and thermal radiation impacts. Therm. Sci..

[22] Sabir Z, Akhtar R, Zhiyu Z, Umar M, Imran A, Wahab HA, Raja MAZ (2019). A computational analysis of two-phase casson nanofluid passing a stretching sheet using chemical reactions and gyrotactic microorganisms. Math. Problems Eng..

[23] Khan S, Ayub A, Shah SZH, Sabir Z, Rashid A, Shoaib M, Ali MR (2023). Analysis of inclined magnetized unsteady cross nanofluid with buoyancy effects and energy loss past over a coated disk. Arab. J. Chem..

[24] Singkibud P, Sabir Z, Al Nuwairan M, Sadat R, Ali MR (2022). Cubic autocatalysis-based activation energy and thermophoretic diffusion effects of steady micro-polar nano-fluid. Microfluidics Nanofluidics.

[25] Ayub A, Sabir Z, Le DN, Aly AA (2021). Nanoscale heat and mass transport of magnetized 3-D chemically radiative hybrid nanofluid with orthogonal/inclined magnetic field along rotating sheet. Case Stud. Therm. Eng..

[26] Ayub A, Sabir Z, Said SB, Baskonus HM, Sadat R, Ali MR (2023). Nature analysis of Cross fluid flow with inclined magnetic dipole. Microsyst. Technol..

[27] Ayub A, Sabir Z, Shah SZH, Wahab HA, Sadat R, Ali MR (2022). Effects of homogeneous-heterogeneous and Lorentz forces on 3-D radiative magnetized cross nanofluid using two rotating disks. Int. Commun. Heat Mass Transf..

[28] Shah SZH, Ayub A, Sabir Z, Adel W, Shah NA, Yook SJ (2021). Insight into the dynamics of time-dependent cross nanofluid on a melting surface subject to cubic autocatalysis. Case Stud. Therm. Eng..

[29] Ayub A, Darvesh A, Altamirano GC, Sabir Z (2021). Nanoscale energy transport of inclined magnetized 3D hybrid nanofluid with Lobatto IIIA scheme. Heat Transfer.

[30] Tarla S, Ali KK, Yilmazer R, Osman MS (2022). The dynamic behaviors of the Radhakrishnan–Kundu–Lakshmanan equation by Jacobi elliptic function expansion technique. Opt. Quant. Electron..

[31] Li R, İlhan OA, Manafian J, Mahmoud KH, Abotaleb M, Kadi A (2022). A mathematical study of the (3+1)-D variable coefficients generalized shallow water wave equation with its application in the interaction between the lump and soliton solutions. Mathematics.

[32] Khani F, Hamedi-Nezhad S (2009). Some new exact solutions of the (2+1)-dimensional variable coefficient Broer-Kaup system using the Exp-function method. Comput. Math. Appl..

[33] Abbagari S, Houwe A, Saliou Y, Douvagaï D, Chu YM, Inc M, Doka SY (2021). Analytical survey of the predator-prey model with fractional derivative order. AIP Adv..

[34] Raza N, Rafiq MH, Kaplan M, Kumar S, Chu YM (2021). The unified method for abundant soliton solutions of local time fractional nonlinear evolution equations. Results Phys..

[35] Zhang JF, Dai CQ, Yang Q, Zhu JM (2005). Variable-coefficient F-expansion method and its application to nonlinear Schrödinger equation. Opt. Commun..

[36] Sirisubtawee S, Thamareerat N, Iatkliang T (2021). Variable coefficient exact solutions for some nonlinear conformable partial differential equations using an auxiliary equation method. Computation.

[37] Ma YL, Li BQ (2022). Kraenkel–Manna–Merle saturated ferromagnetic system: Darboux transformation and loop-like soliton excitations. Chaos Solitons Fract..

[38] Gao XY, Guo YJ, Shan WR, Yuan YQ, Zhang CR, Chen SS (2021). Magneto-optical/ferromagnetic-material computation: Bäcklund transformations, bilinear forms and N solitons for a generalized (3+1)-dimensional variable-coefficient modified Kadomtsev-Petviashvili system. Appl. Math. Lett..

[39] Chu, Y., Shallal, M. A., Mirhosseini-Alizamini, S. M., Rezazadeh, H., Javeed, S., & Baleanu, D. Application of modified extended Tanh technique for solving complex Ginzburg–Landau equation considering Kerr law nonlinearity (2021).

[40] Zhang S, Tong JL, Wang W (2008). A generalized (G'/G)-expansion method for the mKdV equation with variable coefficients. Phys. Lett. A.

[41] Chu Y, Khater M, Hamed YS (2021). Diverse novel analytical and semi-analytical wave solutions of the generalized (2+1)-dimensional shallow water waves model. AIP Adv..

[42] Hosseini K, Sadri K, Mirzazadeh M, Chu YM, Ahmadian A, Pansera BA, Salahshour S (2021). A high-order nonlinear Schrödinger equation with the weak non-local nonlinearity and its optical solitons. Results Phys..

[43] Ramzan M, Chu YM, Rehman H, Saleem M, Park C (2021). Soliton solutions for anti-cubic nonlinearity using three analytical approaches. J. Appl. Anal. Comput.

[44] Chu YM, Fahim MRA, Kundu PR, Islam ME, Akbar MA, Inc M (2021). Extension of the sine-Gordon expansion scheme and parametric effect analysis for higher-dimensional nonlinear evolution equations. J. King Saud Univ.-Sci..

[45] Triki H, Wazwaz AM (2014). Traveling wave solutions for fifth-order KdV type equations with time-dependent coefficients. Commun. Nonlinear Sci. Numer. Simul..

[46] Hu SH, Liu DQ, Ye Y, Li G (2022). Non-local symmetries, consistent Riccati expansion solvability and analytic solutions for the generalized Broer-Kaup system. Pramana.

[47] Kawser MA, Akbar MA, Khan MA (2023). An investigation of the variable coefficients modified KdV equation arising in arterial mechanics by using two expansion techniques. Results Phys..

[48] Arnous AH, Mirzazadeh M, Zhou Q, Moshokoa SP, Biswas A, Belic M (2016). Soliton solutions to resonant nonlinear schrodinger's equation with time-dependent coefficients by modified simple equation method. Optik.

[49] Sheikh MAN, Taher MA, Hossain MM, Akter S (2023). Variable coefficient exact solution of Sharma–Tasso–Olver model by enhanced modified simple equation method. Part. Differ. Equ. Appl. Math..

[50] Rahman Z, Abdeljabbar A, Ali MZ (2022). Novel precise solitary wave solutions of two time fractional nonlinear evolution models via the MSE scheme. Fract. Fract..

[51] Pandir Y, Demiray ST, Bulut H (2016). A new approach for some NLDEs with variable coefficients. Optik.

[52] Ghazanfar S, Ahmed N, Iqbal MS, Akgül A, Bayram M, De la Sen M (2022). Imaging ultrasound propagation using the Westervelt equation by the generalized Kudryashov and modified Kudryashov methods. Appl. Sci..

[53] Akbar MA, Wazwaz AM, Mahmud F, Baleanu D, Roy R, Barman HK, Osman MS (2022). Dynamical behavior of solitons of the perturbed nonlinear Schrödinger equation and microtubules through the generalized Kudryashov scheme. Results Phys..

[54] Mitsotakis DE (2009). Boussinesq systems in two space dimensions over a variable bottom for the generation and propagation of tsunami waves. Math. Comput. Simul..

[55] Kirby JT (2016). Boussinesq models and their application to coastal processes across a wide range of scales. J. Waterway Port Coastal Ocean Eng..

[56] Lynett PJ, Melby JA, Kim DH (2010). An application of Boussinesq modeling to hurricane wave overtopping and inundation. Ocean Eng..

[57] Rashid S, Kaabar MK, Althobaiti A, Alqurashi MS (2023). Constructing analytical estimates of the fuzzy fractional-order Boussinesq model and their application in oceanography. J. Ocean Eng. Sci..

[58] Yao Y, Huang Z, Monismith SG, Lo EY (2012). 1DH Boussinesq modeling of wave transformation over fringing reefs. Ocean Eng..

[59] Ibragimov NH, Ibragimov RN (2010). Internal gravity wave beams as invariant solutions of Boussinesq equations in geophysical fluid dynamics. Commun. Nonlinear Sci. Numer. Simul..

[60] Choi YK, Seo SN, Choi JY, Shi F, Park KS (2019). Wave prediction in a port using a fully nonlinear Boussinesq wave model. Acta Oceanologica Sinica.

[61] Lee, E. S., Violeau, D., Benoit, M., Issa, R., Laurence, D., & Stansby, P. Prediction of wave overtopping on coastal structures by using extended Boussinesq and SPH models. In *Coastal Engineering 2006: (In 5 Volumes)* 4727–4739 (2007).

[62] Kirby, J. T., Noyes, T. J., Guza, R. T., & Elgar, S. Evaluating the low frequency predictions of a Boussinesq wave model: Field cases. In *ISOPE International Ocean and Polar Engineering Conference* ISOPE-I (ISOPE, 2003).

[63] Wazwaz AM (2006). A variety of exact wave solutions with distinct physical structures for the Boussinesq system. Commun. Nonlinear Sci. Numer. Simul..

[64] Shakeel, M. Modified $$(G^{\prime}/G)$$-Expansion Methods for Soliton Solutions of Nonlinear Differential Equations. (*Doctoral dissertation*, *HITEC University Taxila*) (2015).

[65] Chu YM, Rashid S, Karim S, Sultan A (2023). New configurations of the fuzzy fractional differential Boussinesq model with application in ocean engineering and their analysis in statistical theory. CMES-Comput. Model. Eng. Sci..

[66] Olver, P. J. *Applications of Lie Groups to Differential Equations* 107 (Springer, 1993).

